# High cholesterol intake remodels cholesterol turnover and energy homeostasis in Nile tilapia (*Oreochromis niloticus*)

**DOI:** 10.1007/s42995-022-00158-7

**Published:** 2023-02-16

**Authors:** Rui-Xin Li, Ling-Yun Chen, Samwel M. Limbu, Yu-Cheng Qian, Wen-Hao Zhou, Li-Qiao Chen, Yuan Luo, Fang Qiao, Mei-Ling Zhang, Zhen-Yu Du

**Affiliations:** 1 LANEH, School of Life Sciences, East China Normal University, Shanghai, 200241 China; 2 Department of Aquaculture Technology, School of Aquatic Sciences and Fisheries Technology, University of Dar es Salaam, P. O. Box 60091, Dar es Salaam, Tanzania

**Keywords:** Cholesterol metabolism, Metabolic response, Energy metabolism, Gut microbiome

## Abstract

The roles of dietary cholesterol in fish physiology are currently contradictory. The issue reflects the limited studies on the metabolic consequences of cholesterol intake in fish. The present study investigated the metabolic responses to high cholesterol intake in Nile tilapia (*Oreochromis niloticus*), which were fed with four cholesterol-contained diets (0.8, 1.6, 2.4 and 3.2%) and a control diet for eight weeks. All fish-fed cholesterol diets showed increased body weight, but accumulated cholesterol (the peak level was in the 1.6% cholesterol group). Then, we selected 1.6% cholesterol and control diets for further analysis. The high cholesterol diet impaired liver function and reduced mitochondria number in fish. Furthermore, high cholesterol intake triggered protective adaptation via (1) inhibiting endogenous cholesterol synthesis, (2) elevating the expression of genes related to cholesterol esterification and efflux, and (3) promoting chenodeoxycholic acid synthesis and efflux. Accordingly, high cholesterol intake reshaped the fish gut microbiome by increasing the abundance of *Lactobacillus* spp. and *Mycobacterium* spp., both of which are involved in cholesterol and/or bile acids catabolism. Moreover, high cholesterol intake inhibited lipid catabolic activities through mitochondrial β-oxidation, and lysosome-mediated lipophagy, and depressed insulin signaling sensitivity. Protein catabolism was elevated as a compulsory response to maintain energy homeostasis. Therefore, although high cholesterol intake promoted growth, it led to metabolic disorders in fish. For the first time, this study provides evidence for the systemic metabolic response to high cholesterol intake in fish. This knowledge contributes to an understanding of the metabolic syndromes caused by high cholesterol intake or deposition in fish.

**Supplementary Information:**

The online version contains supplementary material available at 10.1007/s42995-022-00158-7.

## Introduction

Cholesterol is an essential cellular structural component, which serves as a precursor for the biosynthesis of bile acids, vitamin D and steroid hormones (Goedeke and Fernández-Hernando 2012). The tight relationship between cholesterol and cardiovascular disease has been recognized for a long time (Berger and Raman 2015). Moreover, cholesterol plays an important function in regulating energy metabolism in mammals (Castellano and Thelen 2017; Chen et al. 2019). However, high cholesterol levels are associated with an increased risk of metabolic syndromes, including nonalcoholic fatty liver disease and obesity (Chen et al. 2019). These are always characterized by excessive accumulation of triglyceride (TG) and cholesterol in liver (Min et al. 2012). Indeed, the high dietary cholesterol-induced lipogenesis pathway is associated with liver X receptor (LXR)-activated sterol regulatory element-binding proteins 1c (SREBP1c) effects (Han et al. 2009, 2013). High cholesterol synthesis is a common symptom in type 2 diabetes as evidenced by a direct link between elevated serum cholesterol and reduced insulin secretion (Hao et al. 2007; Stranberg et al. 1996). Therefore, to maintain cellular cholesterol homeostasis, mammals tightly control cholesterol biosynthesis, uptake, transport, efflux and esterification (Luo et al. 2020).

Cholesterol is obtained either exogenously from diet intake or by endogenous biosynthesis in animals, with the latter being the major source. Fish were previously believed to grow normally and healthily without exogenous cholesterol supplementation in diets (Jobling 2012; Sealey et al. 2001). This was because they have a similar cholesterol synthesis pathway as mammals, and could regulate cholesterol homeostasis based on cellular sterol levels (Kortner et al. 2013; Zhu et al. 2018a). Evidently, cholesterol biosynthetic genes and their transcriptional factors were up-regulated in Atlantic salmon (*Salmo salar*) after dietary fishmeal or fish oil was replaced by vegetable ingredients (without cholesterol inclusion) (Kortner et al. 2013; Leaver et al. 2008). Similarly, rainbow trout (*Oncorhynchus mykiss*) increased cholesterol synthesis and limited cholesterol efflux through transcriptional regulators, such as *srebp-2*, *lxrα* and MicroRNA-223 (*miR-223*), without dietary cholesterol supplementation (Zhu et al. 2018a). However, previous studies reported that dietary cholesterol supplementation resulted in beneficial effects in some fish species. For example, dietary cholesterol supplementation (1.0–1.5%) in soybean meal diet as a protein source increased body weight in turbot (*Scophthalmus maximus*) (Yun et al. 2011) and channel catfish (*Ictalurus punctatus*) (Twibell and Wilson 2004). Notably, the increased body weight was accompanied by higher plasma and hepatic cholesterol in channel catfish (Twibell and Wilson 2004) similar to single-cell level in human patients with cancerous tissues (Yue et al. 2014). On the contrary, high dietary cholesterol intake inhibited a key gene involved in cholesterol synthesis (3-hydroxy-3-methyl-glutaryl-coenzyme A reductase, *hmgcr*) and promoted bile acid synthesis-related gene (cholesterol 7 alpha-hydroxylase, *cyp7a1*) in juvenile turbot (Zhu et al. 2018b). Moreover, a previous study indicated that dietary cholesterol inclusion increased the incidence of the arteriosclerotic lesion and fatty liver in fish fed on either fishmeal-based or soy-based protein diets (Deng et al. 2010). In addition, cholesterol accumulation has been reported frequently in high-energy diet-fed fish, accompanied by metabolic liver diseases (Chen et al. 2022; He et al. 2021; Tang et al. 2019; Tao et al. 2018; Xu et al. 2022). For example, high-carbohydrate diet induced the accumulation of hepatic TG and total cholesterol, causing liver damage in Nile tilapia (Xu et al. 2022). Similarly, the accumulated TG and total cholesterol were observed in the liver of Nile tilapia fed high-fat diet, which may well weaken hepatic antioxidant capacity (Tao et al. 2018). Therefore, the roles of dietary cholesterol in fish physiology are currently contradictory. The issue reflects the limited studies on the metabolic consequences of high cholesterol intake in fish. Further studies are required on metabolic responses to understand the exact nutritional roles of cholesterol in fish.

Nile tilapia (*Oreochromis niloticus*) is a widely cultured economically important fish species. Recently, Nile tilapia has been used as an ideal animal model for metabolism research because of its rapid growth rate and availability of genomic information (Guyon et al. 2012; Ning et al. 2016). The present study aimed to study the systemic metabolic responses of fish to cholesterol intake.

## Results

### Dietary cholesterol intake increased growth but caused cholesterol accumulation and impaired liver

**Fig. 1:**
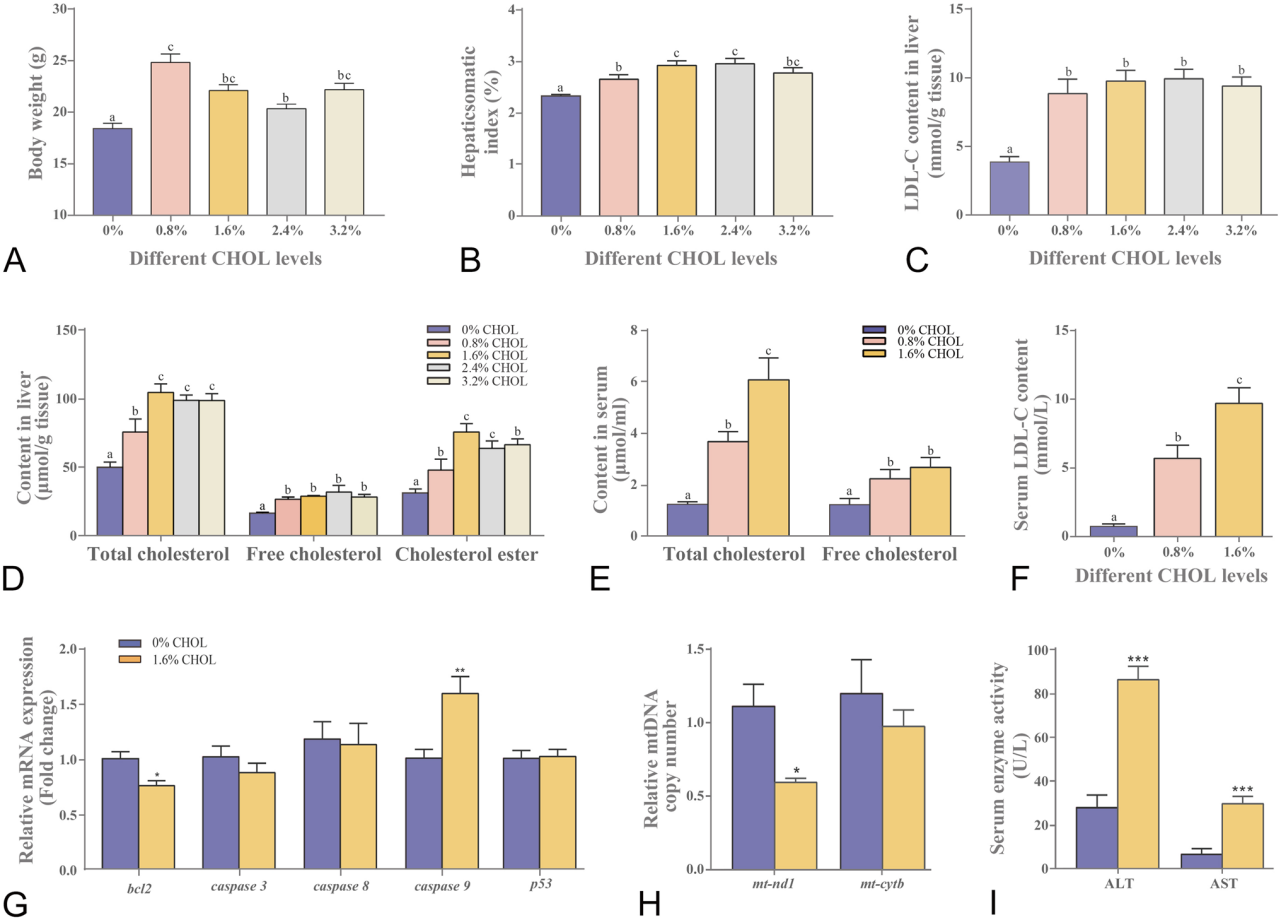
High cholesterol intake increased body weight but caused serum and hepatic cholesterol accumulation and impaired liver in Nile tilapia. **A** Body weight; **B** Hepatosomatic index; **C** LDL-C; and **D** Cholesterol contents in liver; **E** Total cholesterol and free cholesterol; and **F** LDL-C levels in serum; **G** mRNA expression of apoptosis genes; **H** Mitochondrial copy number in liver; **I** ALT and AST enzyme activities in serum. Data are represented as mean ± SD (body weight: *n* = 30; hepatosomatic index: *n* = 12; liver and serum TC, FC, CE and LDL-C contents; serum ALT and AST; mitochondrial copy number in liver; gene expression: *n* = 6). Data (**A**–**F**) were subjected to one-way analysis of variance (ANOVA) followed by Tukey’s multiple range test. Bars assigned with different superscripts are significantly different (*P* < 0.05). *P < 0.05, **P < 0.01 and ***P < 0.001 indicate significant difference (independent *t *test) between 0 and 1.6% dietary cholesterol groups (**G**, **H** and **I**); *CHOL* cholesterol, *TC* total cholesterol, *FC* free cholesterol, *CE* cholesterol ester, *LDL-C* low-density lipoprotein cholesterol, *ALT* alanine aminotransferase, *AST* aspartate aminotransferase, *mt-nd1* mitochondrial NADH dehydrogenase 1, *mt-cytb* mitochondrial cytochrome b, *bcl2* B-cell lymphoma 2, *caspase-3/8/9* cysteine-aspartic acid protease-3/8/9, *p53* tumor protein p53

**Fig. 2:**
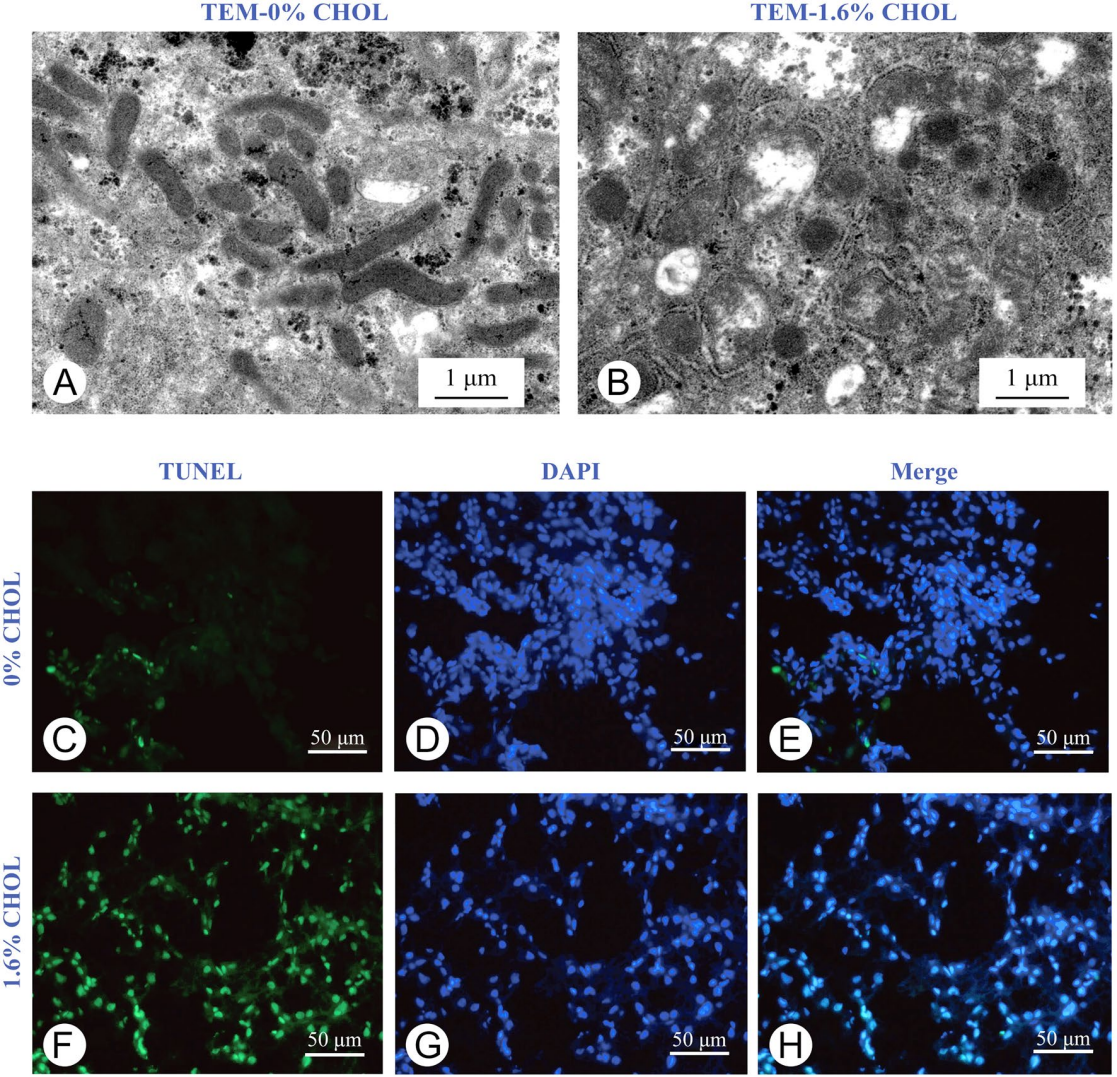
High cholesterol intake induced liver mitochondrial damage and cell apoptosis in Nile tilapia. **A**, **B** Transmission electron microscope of mitochondria; **C**–**H** TUNEL staining (the lower) images in liver. Data are represented as mean ± SD (transmission electron microscope and TUNEL staining images of liver: *n* = 4)

We first established the high-cholesterol Nile tilapia model by feeding with different dietary levels of cholesterol for eight weeks. During the feeding trial, all fish ate comparable feeds. All dietary cholesterol supplementation at 0.8–3.2% led to increased body weight (Fig. 1A) and hepatosomatic index (Fig. 1B). Furthermore, as compared with the control, fish fed with 1.6% dietary cholesterol had higher hepatic LDL-C (Fig. 1C) and TC, FC, CE (Fig. 1D) contents that peaked among the different cholesterol treatments. Therefore, 1.6% dietary cholesterol was sufficient for establishing the high-cholesterol model. Similarly, the fish fed on 1.6% dietary cholesterol increased significantly the concentrations of TC, FC (Fig. 1E) and LDL-C (Fig. 1F) in the serum, although there was only slightly increased TC content in adipose (Supplementary Fig. S1A) and muscle tissues (Supplementary Fig. S1B) but not in the intestine (Supplementary Fig. S1C). The FC accumulation was not significantly different in adipose (Supplementary Fig. S1D), muscle (Supplementary Fig. S1E) and intestine tissues (Supplementary Fig. S1F). Moreover, the fish fed on high cholesterol diet decreased mitochondria number (Fig. 1H and 2A) and increased cell apoptosis (Fig. 2B). Accordingly, the up-regulated intrinsic apoptosis caspase-9 and down-regulated anti-apoptosis B-cell lymphoma-2 (*bcl2*) gene expressions were found in the liver of the high cholesterol diet-fed fish (Fig. 1G). But the extrinsic apoptosis caspase-8 gene expression level was comparable between the high cholesterol and control groups (Fig. 1G). Thus, higher serum ALT and AST enzyme activities, which indicated impaired liver health, were observed in fish fed on high cholesterol diet (Fig. 1I). In the following assays, the fish fed on 1.6% cholesterol diet were chosen to explore the metabolic responses to high cholesterol intake.

### High cholesterol intake inhibited hepatic cholesterol synthesis and promoted bile acid turnover

**Fig. 3:**
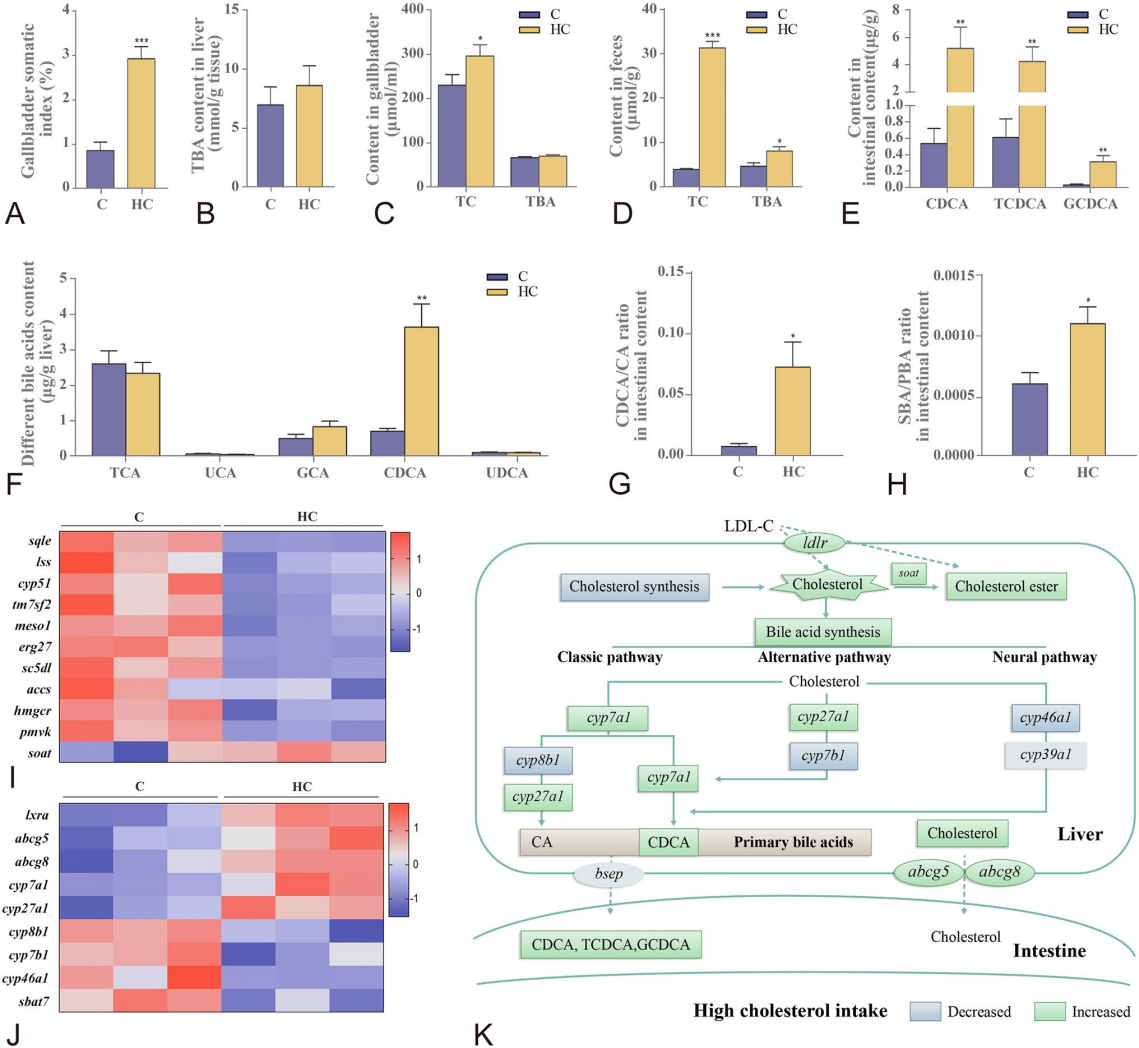
High cholesterol intake inhibited hepatic cholesterol synthesis, elevated selective bile acid synthesis and efflux in Nile tilapia. **A** Gallbladder somatic index; **B** Liver TBA content; **C** Gallbladder TC and TBA contents; **D** Feces TC and TBA contents; **E** CDCA, TCDCA and GCDCA in intestinal content; **F** Different bile acids content in liver; **G**, **H** CDCA/CA and SBA/PBA ratios in intestinal content; **I** mRNA expression of hepatic cholesterol synthesis and esterification genes; **J** mRNA expression of hepatic bile acids metabolism genes; **K** Schematic on high cholesterol intake triggers protective adaptation via inhibiting cholesterol synthesis and promoting bile acids turnover. Data are represented as mean ± SD (gallbladder somatic index: *n* = 12; gallbladder/feces TC contents and liver/gallbladder/feces/intestinal content bile acids contents: *n* = 6; transcriptome heatmap, *n* = 9; *P < 0.05, **P < 0.01 and ***P < 0.001 indicates significant difference (independent *t* test) between HC and C treatments. *C* control diet, *HC* high cholesterol diet, *TC* total cholesterol, *TCA* taurocholic acid sodium salt, *UCA* ursocholic acid, *GCA* sodium glycocholate hydrate, *CDCA* chenodeoxycholic acid, *UDCA* ursodeoxycholic acid, *TCDCA* taurochenodeoxycholic acid, *GCDCA* glycochenodeoxycholic acid sodium salt, *SBA/PBA* secondary bile acids/primary bile acids, *sqle* squalene monooxygenase, *lss* lanosterol synthase, *cyp51* lanosterol 14-alpha-demethylase, *tm7sf2* delta14-sterol reductase, *msmo1* methylsterol monooxygenase 1, *erg27* 3-keto steroid reductase, *scd5dl* Delta7-sterol 5-desaturase, *aact* acetyl-CoA C-acetyltransferase, *hmgcr* 3-hydroxy-3-methylglutaryl-Coenzyme A reductase, *pmvk* phosphomevalonate kinase, *soat* sterol O-acyltransferase, *lxrɑ* liver x receptor ɑ, *abcg5/8* ATP-binding cassette, subfamily G, member 5/8, *cyp7a1* cholesterol 7alpha-hydroxylase, *cyp27a1* cytochrome P450 family 27 subfamily A member 1, *cyp8b1* sterol 12a-hydroxylase, *cyp7b1* cytochrome P450 family 7 subfamily B member 1, *cyp46a1* cytochrome P450 family 46 subfamily A member 1, *sbat7* sodium/bile acid cotransporter 7

High cholesterol accumulation was observed mainly in the liver of fish fed on cholesterol-supplemented diet. Accordingly, the fish fed on high cholesterol level developed enlarged gallbladders (Fig. 3A). Furthermore, Nile tilapia fed on high cholesterol diet increased cholesterol contents in the gallbladder (Fig. 3C) and feces (Fig. 3D). Correspondingly, the fish fed on high cholesterol diet inhibited significantly the genes related to cholesterol synthesis (such as *hmgcr*; squalene epoxidase, *sqle*; and lanosterol synthase, *lss*, Fig. 3I), whereas the genes related to cholesterol esterification (sterol O-acyltransferase, *soat*) and cholesterol efflux into the gallbladder (ATP-binding cassette sub-family G member 5 and 8, *abcg5* and *abcg8*) were upregulated significantly in the liver (Fig. 3J). In addition, the fish fed on high cholesterol diet had higher levels of total bile acids in the feces (Fig. 3D), but not in the liver (Fig. 3B) and gallbladder (Fig. 3C). Moreover, Nile tilapia fed on high cholesterol diet increased significantly the chenodeoxycholic acid (CDCA) content in the liver among several main primary bile acids (Fig. 3F). Correspondingly, the fish fed on high cholesterol diet increased significantly the expression of genes related to CDCA synthesis (*cyp7a1*, cholesterol 7alpha-hydroxylase), whereas the expression of sterol 12a-hydroxylase (*cyp8b1*) gene, which is related to cholic acid (CA) synthesis as another primary bile acid, was depressed in the liver (Fig. 3J). In addition, the fish fed on high cholesterol diet decreased significantly the expression levels of cytochrome P450 family 7 subfamily B member 1 (*cyp7b1*) and cytochrome P450 family 46 subfamily A member 1 (*cyp46a1*) genes, which are key enzymes in the alternative and neutral pathways for bile acids synthesis in the liver (Fig. 3J). Thus, the hepatic primary bile acid CDCA was synthesized mainly through the classical pathway, whereas the CA synthesis pathway was blocked in the high-cholesterol fish (Fig. 3K). Furthermore, Nile tilapia fed with the high cholesterol diet increased significantly the contents of CDCA and its conjugated forms, i.e. taurochenodeoxycholic acid (TCDCA) and glycochenodeoxycholic acid sodium salt (GCDCA; Fig. 3E), and CDCA/CA ratio in the intestinal content (Fig. 3G). Moreover, the fish fed on high cholesterol diet had significantly higher secondary bile acids/primary bile acids ratio (SBA/PBA), which are the markers of the ability to convert primary bile acids to secondary bile acids in the intestine (Fig. 3H). These results suggested that the inhibited cholesterol synthesis, elevated selective bile acid synthesis and efflux were adaptive mechanisms towards high cholesterol intake (Fig. 3K).

### High cholesterol intake caused hepatic lipid deposition by inhibiting lipid catabolism

**Fig. 4:**
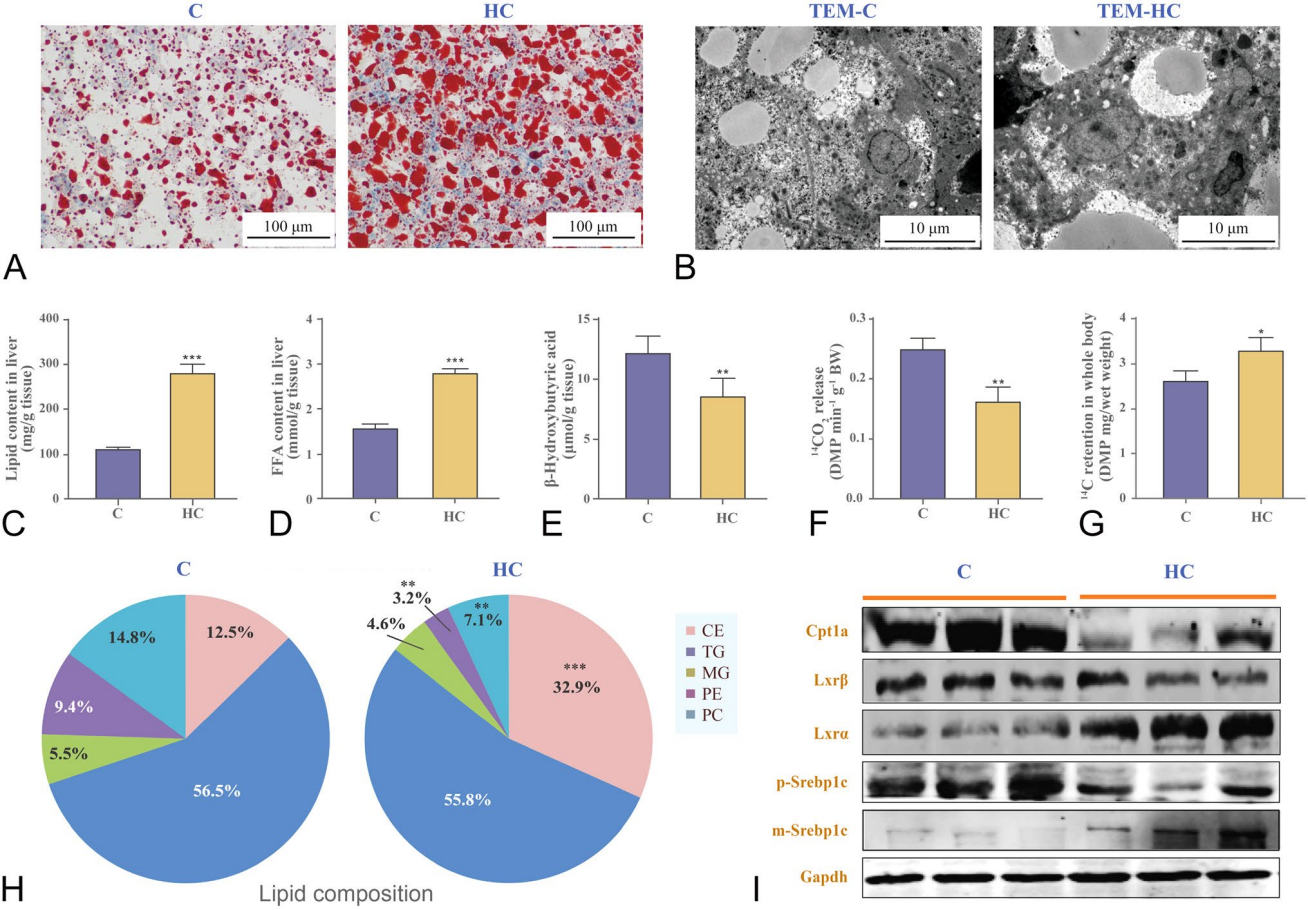
High cholesterol intake caused hepatic lipid deposition by inhibiting lipid catabolism in Nile tilapia. **A** Oil Red staining; **B** Transmission electron microscope images of liver; **C** Liver lipid content; **D** Liver FFA content; **E** Liver β-Hydroxybutyric acid content; **F**, **G** ^14^CO_2_ release and ^14^C retention in whole body after the [1-^14^C]-PA oxidation test; **H** Liver lipid composition; **I** Protein expressions of Cpt1a, Lxr and Srebp1c. Data are represented as mean ± SD (liver lipid and FFA, β-hydroxybutyric acid contents, gene expression and ^14^CO_2_ release and ^14^C retention in whole body: *n* = 6; Oil Red staining and transmission electron microscope images of liver: *n* = 4; protein expression: *n* = 6). *P < 0.05, **P < 0.01 and ***P < 0.001 indicate significant difference (independent *t *test) between HC and C treatments. *C* control diet, *HC* high cholesterol diet, *CE* cholesterol ester, *TG* triglyceride, *MG* monoacylglycerol, *PE* phosphatidylethanolamine, *PC* phosphatidylcholine, *PA* palmitic acid, *FFA* free fatty acid, *Cpt1a* carnitine palmitoyl transferase 1a, *Lxrα/β* liver x receptorα/β, *p/m-Srebp1c* precursor/mature form of sterol regulatory element binding protein 1c, *Gapdh* glyceraldehyde-3-phosphate dehydrogenase

**Fig. 5:**
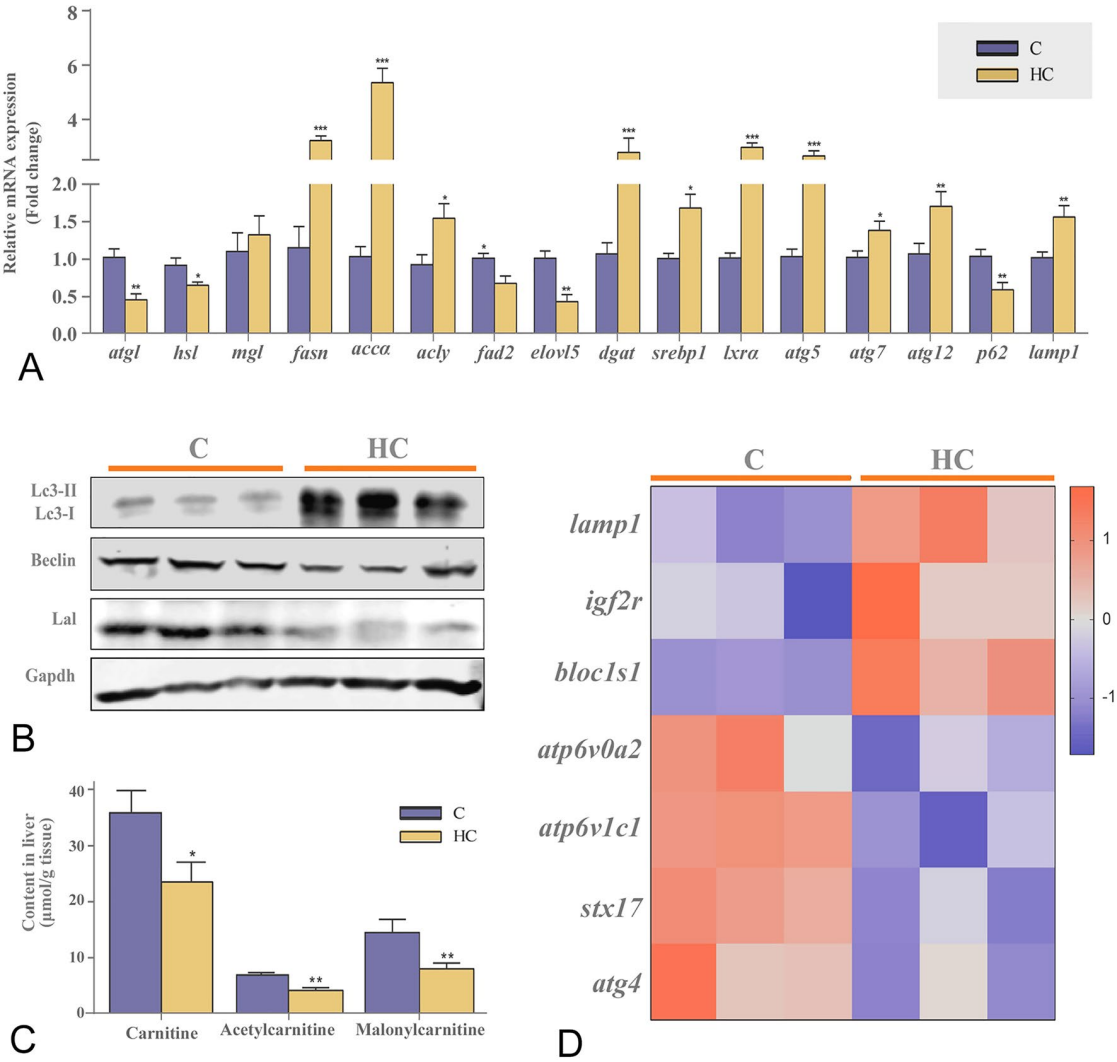
High cholesterol intake inhibited lipolysis, lipophagy and mitochondrial fatty acid β-oxidation but stimulated lipogenesis pathways. **A** mRNA expression of hepatic lipid metabolism genes; **B** Protein expressions of Lc3 I/II, Beclin and Lal in liver; **C** Liver carnitine, acetlycarnitine and malonylcarnitine contents; **D** mRNA expression of hepatic autophagy genes. Data are represented as mean ± SD (liver carnitine, acetlycarnitine and malonylcarnitine contents, gene expression: *n* = 6; protein expression: *n* = 6; transcriptome heatmap: *n* = 9). *P < 0.05, **P < 0.01 and ***P < 0.001 indicate significant difference (independent *t *test) between HC and C treatments. *C* control diet, *HC* high cholesterol diet, *atgl* adipose triglyceride lipase, *hsl* hormone-sensitive lipase, *mgl* monoacylglycerol lipase, *fasn* fatty acid synthase, *accɑ* acetyl-CoA carboxylase *a*, *acly* ATP citrate lyase, *dgat* diacylglycerol acyltransferases, *Lc3* microtubule-associated protein light chain 3, *Lal* lysosomal acid lipase, *Gapdh* glyceraldehyde-3-phosphate dehydrogenase, *atg5/7/12* autophagy-related genes 5/7/12, *p62* sequestosome 1, *lamp1* lysosomal associated membrane protein 1, *igf2r* insulin like growth factor 2 receptor, *bloc1s1* biogenesis of lysosomal organelles complex 1 subunit 1, *atp6v0a2* ATPase H + transporting V0 subunit A2; *atp6v1c1*, V-type proton ATPase subunit C1, *stx17*, Syntaxin 17

Nile tilapia fed on high cholesterol diet developed enlarged livers (Supplementary Fig. S2A), and had severe lipid deposition, including total lipid (Fig. 4A, C) and FFA (Fig. 4D). The fish fed on high cholesterol diet had significantly higher cholesterol ester, but lower phospholipids (manly phosphatidylcholine, PC and phosphatidylethanolamine, PE) than the control (Fig. 4H). Indeed, the high-cholesterol diet caused the accumulation of liver TG insofar as the higher total lipid content and the unchanged level of TG in total lipid were found in the livers of high-cholesterol fish (Fig. 4C, H). Furthermore, Nile tilapia fed on the high cholesterol diet decreased the mRNA expression of genes related to lipolysis, such as adipose triglyceride lipase (*atgl*) and hormone-sensitive lipase (*hsl*), but increased lipogenesis genes, i.e. fatty acid synthase (*fasn*), ATP citrate lyase (*acly*), acetyl-CoA carboxylase alpha (*accα*), acyl-CoA:diacylglycerol acyltransferase (*dgat*) and *srebp1* in the liver (Fig. 5A). In addition, high cholesterol diet intake upregulated m-Srebp1 (mature and active forms of SREBP1) and Lxrα protein expressions, but decreased Cpt1a protein expression in the liver (Fig. 4I and Supplementary Fig. S2B). Moreover, Nile tilapia fed on the high cholesterol diet had much larger lipid droplets in hepatocytes, which were enriched in the lysosomal lumen but not broken down compared to those in the control (Fig. 4B). Furthermore, we observed significant up-regulation of autophagy marker protein Lc3-II (microtubule-associated protein light chain 3- phosphatidylethanolamine conjugate; Fig. 5B and Supplementary Fig. S2C), mRNA expression of autophagosome formation genes (autophagy-related genes 5/7/12: *atg5*, *atg7* and *atg12*; Fig. 5A) and lysosome biosynthesis-related genes (*lamp1*, lysosomal associated membrane protein 1; *igf2*, insulin like growth factor 2 receptor; *bloc1s1*, biogenesis of lysosomal organelles complex 1 subunit 1, Fig. 5D) in the fish fed on the high cholesterol diet, whereas beclin and lysosomal acid lipase (Lal) proteins (Fig. 5B and Supplementary Fig. S2C), autophagosome-lysosome fusion (*stx17*, syntaxin 17), lysosome acidification (*atp6v0a2*, ATPase H + transporting V0 subunit A2; *atp6v1c1*, V-type proton ATPase subunit C1) related gene expressions declined significantly in the liver (Fig. 5D). Moreover, Nile tilapia fed on the high cholesterol diet decreased significantly the carnitine, acetylcarnitine and malonycarnitine (Fig. 5C) and β-hydroxybutyric acid contents (Fig. 4E). These are biochemical markers for mitochondrial β-oxidation in the liver. The fish fed on the high cholesterol diet released less ^14^CO_2_ (Fig. 4F) but had higher ^14^C-lipid retention than the controls (Fig. 4G) after the [1-^14^C]-PA oxidation tracking test. These data indicated that the high-cholesterol diet promoted lipid deposition by inhibiting lipolysis, lipophagy and mitochondrial fatty acid β-oxidation.

### Cholesterol overload impaired insulin signaling pathway and inhibited glucose oxidation

**Fig. 6:**
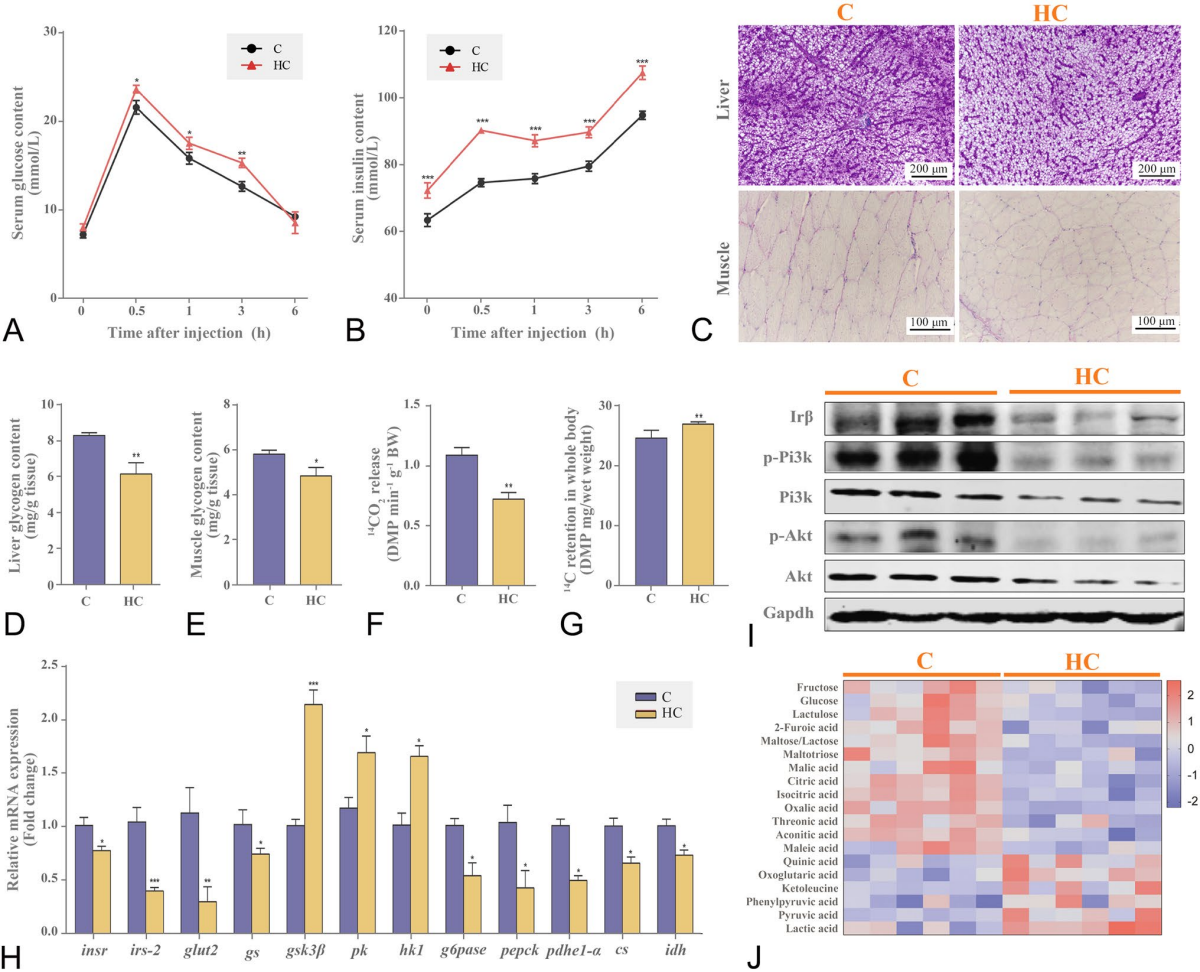
Cholesterol overload impaired insulin signaling pathway and inhibited glucose oxidation in the liver of Nile tilapia. **A**, **B** Serum glucose and insulin contents in GTT; **C** PAS staining in liver and muscle; **D**, **E** Glycogen contents in liver and muscle; **F**, **G** ^14^CO_2_ release and ^14^C retention in whole body after the [1-^14^C]-glucose oxidation test; **H** mRNA expression of hepatic glucose metabolism and TCA genes; **I** Protein expression of insulin pathway; **J** Metabolites associated with glucose metabolism;. Data are represented as mean ± SD (serum glucose and insulin contents in GTT, glycogen contents in liver and muscle, gene expression and ^14^CO_2_ release and ^14^C retention in whole body: *n* = 6; PAS staining images of liver and muscle: *n* = 4; Protein expression: *n* = 6; metabolomics heatmap: *n* = 12). *P < 0.05, **P < 0.01 and ***P < 0.001 indicate significant difference (independent *t* test) between HC and C groups. *C* control diet, *HC* high cholesterol diet, *GTT* glucose tolerance test, *PAS* periodic acid-schiffs, *TCA* tricarboxylic acid cycle, *insr* insulin receptor, *irs-2* insulin receptor substrate 2, *glut2* glucose transporter 2, *gs* glycogen synthetase, *gsk3β* glycogen synthase kinase-3*β*, *pk* pyruvate kinase, *hk1* hexokinase 1, *g6pase* glucose-6-phosphatase, *pepck* phosphoenolpyruvate carboxykinase, *pdhe1-a* pyruvate dehydrogenase E1 component subunit alpha, *cs* citrate synthase, *idh* isocitrate dehydrogenase, *Irβ* insulin receptor beta, *Pi3k* phosphoinositide 3-kinase, *Akt* serine/threonine kinase, *Gapdh* glyceraldehyde-3-phosphate dehydrogenase

Nile tilapia fed with the high cholesterol diet showed weaker glucose clearance (Fig. 6A) and insulin sensitivity (Fig. 6B) than the control. The fish fed on the high cholesterol diet led to reduced glycogen contents (Fig. 6C) in the liver (Fig. 6D) and muscle (Fig. 6E), accompanied by the inhibited expression of Irβ-Pi3k-Akt (Irβ, insulin receptor beta; Pi3k, phosphoinositide 3-kinase; Akt, serine/threonine kinase) signaling proteins (Fig. 6I and supplementary Fig. S3A) and genes (*insr*, insulin receptor; *irs-2*, insulin receptor substrate 2; g*lut2*, glucose transporter 2; *gs*, glycogen synthetase) (Fig. 6H). In addition, fish fed with the high cholesterol diet upregulated significantly glycolytic genes (*hk1*, hexokinase 1; *pk*, pyruvate kinase), but downregulated gluconeogenic genes (*g6pase*, glucose-6-phosphatase; *pepck*, phosphoenolpyruvate carboxykinase) in the liver (Fig. 6H). It was observed that Nile tilapia fed with the high cholesterol diet led to significant reductions in glucose, fructose, lactose and other simple carbohydrates in the liver (Fig. 6J). Furthermore, Nile tilapia fed with the high cholesterol diet led to increased lactate and pyruvate levels (Fig. 6J), and the increased expression of lactate dehydrogenase-A (*ldha*) and alcohol dehydrogenase (*adh*) genes, but decreased pyruvate dehydrogenase E1 subunit (*pdhe1*) and mitochondrial pyruvate carrier 2 (*mcp2*) genes in the liver (Supplementary Fig. S3B). The fish fed with the high cholesterol diet decreased the tricarboxylic acid cycle (TCA) related metabolites, including malic acid, citric acid and isocitrate contents (Fig. 6J). In addition, the gene expression levels of key enzymes (*cs*, citrate synthase; *idh*, isocitrate dehydrogenase) were downregulated significantly in the liver (Fig. 6H). Furthermore, fish fed with a high cholesterol diet increased significantly a-ketoglutarate, which is a TCA intermediate metabolite from amino acids in the liver (Fig. 6J). After the [1-^14^C] glucose oxidation test, Nile tilapia fed with the high cholesterol diet released less ^14^CO_2_ (Fig. 6F) but had higher ^14^C-glucose retention (Fig. 6G) than those in the control. These results indicated that the fish fed on the high-cholesterol diet had low insulin sensitivity and did not preferentially oxidize glucose for energy production.

### Cholesterol overload inhibited protein synthesis and promoted protein catabolism for energy supply

**Fig. 7:**
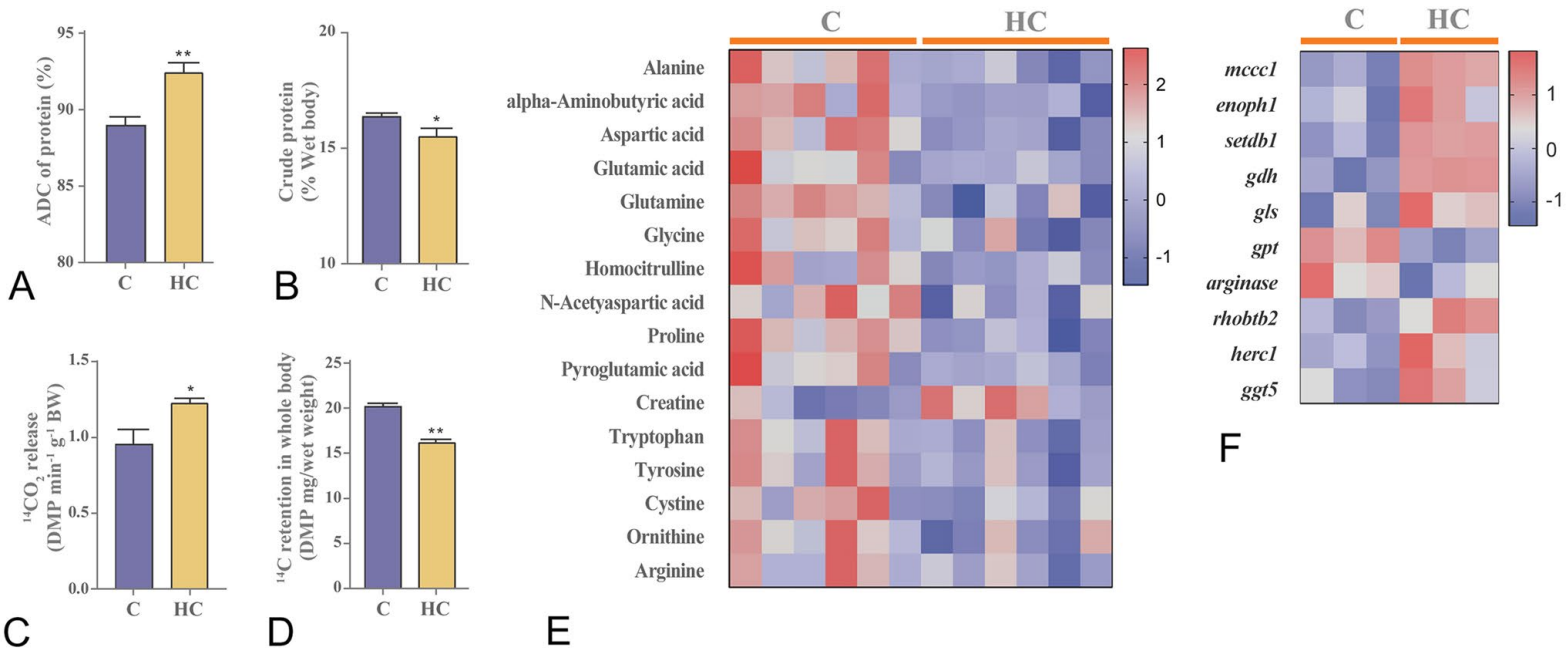
Cholesterol overload inhibited protein synthesis and promoted protein catabolism for energy supply. **A** Dietary ADC of protein; **B** Crude protein in whole body; **C**, **D** ^14^CO_2_ release and ^14^C retention in whole body after the [1-^14^C]-amino acid oxidation test; **E** Different amino acid content in liver; **F** Gene expression of amino acid catabolism in liver. Data are represented as mean ± SD (dietary ADC of protein, crude protein in whole body, ^14^CO_2_ release and.^14^C retention in whole body: *n* = 6; protein expression: *n* = 6; transcriptome heatmap: *n* = 9; and metabolomics heatmap: *n* = 12). *P < 0.05, **P < 0.01 and ***P < 0.001 indicate significant difference (independent *t* test) between HC and C groups. *C* control diet, *HC* high cholesterol diet, *ADC* apparent digestibility coefficient, *mccc1* methylcrotonoyl-CoA carboxylase 1, *enoph1* enolase-phosphatase E1 isoform, *setdb1* histone-lysine N-methyltransferase, *gdh* glutamate dehydrogenase, *gls* glutaminase, *gpt* glutamic–pyruvic transaminase, *rhobtb2* rho related BTB domain containing 2, *herc1* HECT And RLD domain containing E3 ubiquitin protein ligase family member 1, *ggt5* gamma-glutamyltransferase 5

Surprisingly, Nile tilapia fed with the high cholesterol diet had significantly higher protein ADC than the control (Fig. 7A). However, fish fed with this diet decreased total body protein content (Fig. 7B). In addition, fish fed on this high cholesterol diet led to significant decreases in most amino acids (Fig. 7E), whereas there was significant up-regulation in the expression of genes related to amino acid catabolism (Fig. 7F). Furthermore, fish fed with the high cholesterol diet downregulated significantly the expression levels of p-mTorc1 and p-S6 proteins in the liver (Supplementary Fig. S3C). Moreover, Nile tilapia fed with this diet released a higher amount of ^14^CO_2_ (Fig. 7C), but had lower ^14^C-protein storage than those in the control after intraperitoneal injection of [l-^14^C (U)]-AA (Fig. 7D). These data indicated that the high cholesterol intake promoted protein absorption and catabolism for energy supply in fish.

### High cholesterol intake reshaped gut microbiome

**Fig. 8:**
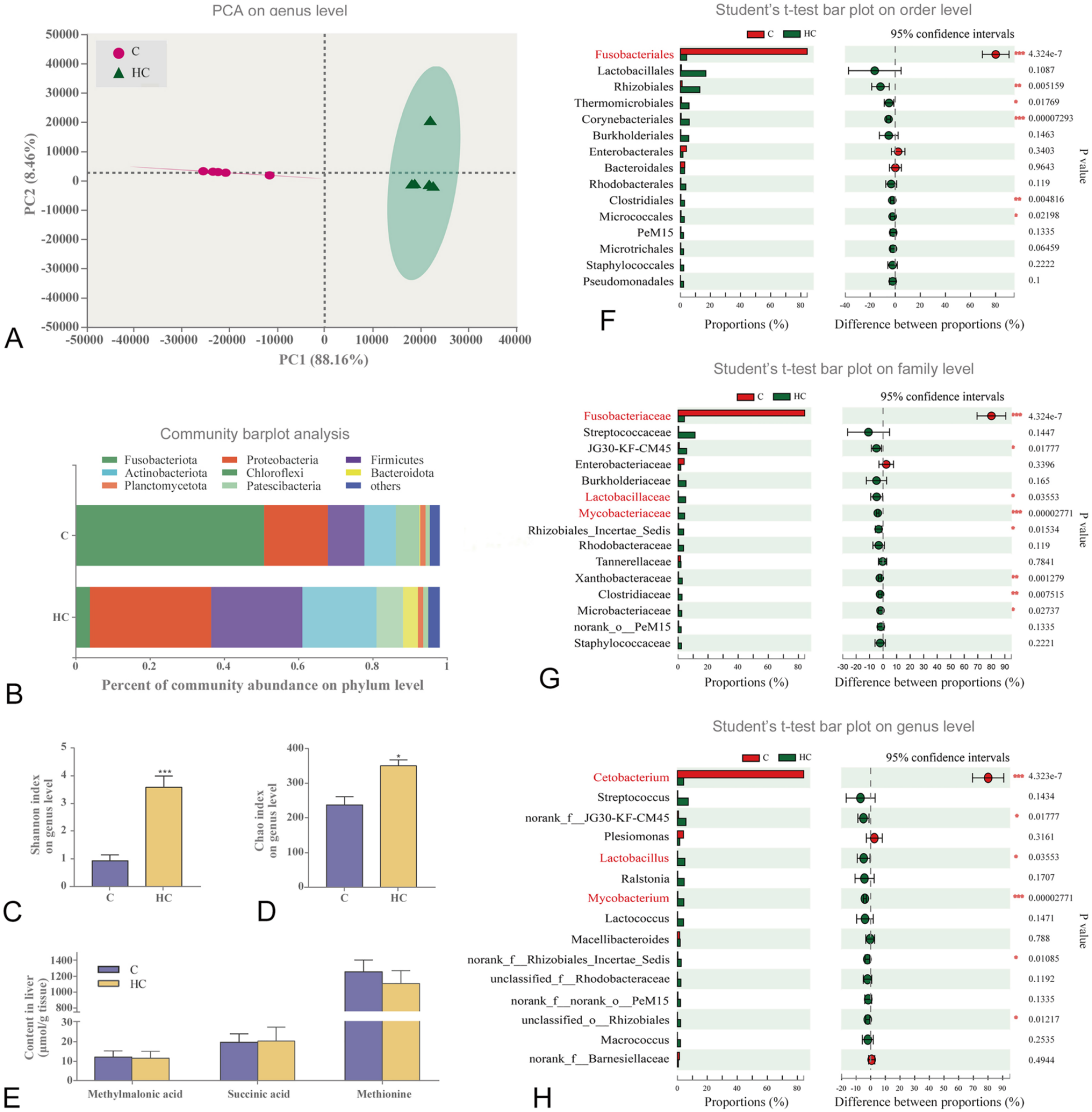
High cholesterol intake reshaped the gut microbiome. **A** PCA analysis on the genus level; **B** composition of gut microbiota at the phylum level; **C**, **D** Shannon index and Chao index at the genus level; **E** Methylmalonic acid, succinic acid and methionine contents in liver; **F**–**H** community abundance of the dominant bacteria in order, family and genus in gut. Data are represented as mean ± SD (gut microbiota analysis and liver methylmalonic acid, succinic acid and methionine: *n* = 6). *P < 0.05, **P < 0.01 and ***P < 0.001 indicate significant difference (independent *t *test) between HC and C groups. *C* control diet, *HC* high cholesterol diet

The intestinal bacterial composition was different between the fish fed on high cholesterol and the control (Fig. 8A). Feeding Nile tilapia with the high cholesterol diet decreased significantly the abundance of the *Fusobacteria* phylum, but increased significantly *Proteobacteria*, *Firmicutes*, *Actinobacteria* and *Bacteroides* phyla (Fig. 8B). In addition, the high cholesterol diet increased intestinal microbial diversity based on the Shannon index (Fig. 8C) and Chao index at the genus level (Fig. 8D). Further analysis of different intestinal microbiomes at the genus level revealed that the fish fed with the high cholesterol diet increased significantly the abundance of *Lactobacillus* spp. and *Mycobacterium* spp., which are involved in cholesterol and/or bile acids catabolism (Fig. 8F–H). However, Nile tilapia fed with the high cholesterol diet significantly reduced the abundance of *Cetobacterium* spp., which mainly produces vitamin B_12_ and is thereby involved in protein anabolism (methionine synthesis) and oxidative phosphorylation (the conversion of methylmalonic acid to succinic acid; Fig. 8E). However, fish fed on the high cholesterol and control diets did not reveal any significant difference in methylmalonic acid, succinic acid and methionine (Fig. 8E) contents in the liver, suggesting the decreased abundance of *Cetobacterium* spp. may have only marginal effects on protein metabolism. In general, these results indicated that high cholesterol intake induced adaptive changes in the gut microbiota composition.

## Discussion

### High cholesterol intake triggered protective adaptation via remodeling cholesterol synthesis and turnover

Fish obtain cholesterol either from the diet or de novo synthesis. Cholesterol homeostasis in organisms is achieved through the regulation of its biosynthesis, uptake, transport, efflux and esterification (Sheridan 1988). Cholesterol metabolism in organisms is tightly controlled by several feedback mechanisms to avoid its excess accumulation, including regulation by transcription factors such as LXRs (Luo et al. 2020). LXRs, as cholesterol sensors, are activated by endogenous oxysterols (oxidized derivatives of cholesterol), which mediate cholesterol efflux and bile acid synthesis genes to maintain cellular cholesterol homeostasis (Sallam et al. 2016). In the present study, the cholesterol-stimulated Lxrα protein expression was in accordance with the activated hepatic cholesterol efflux and bile acid synthesis in fish fed with the high-cholesterol diet. Subsequently, the excess cholesterol inhibited the expression of genes related to cholesterol synthesis in the liver of fish fed with this diet. Cholesterol elimination has been reported to occur in the liver mainly through direct cholesterol and indirect bile acid excretion into the gallbladder and intestine (Dietschy 1984). In the present study, cholesterol accumulation was observed in most tissues especially in the liver of fish fed with the high cholesterol diet. Furthermore, high cholesterol content was found in the gallbladder and feces concurrent with the upregulation of hepatic cholesterol efflux-related genes in the fish fed with the high cholesterol diet indicating the stimulated excretion of cholesterol into the gallbladder and intestine.

**Fig. 9:**
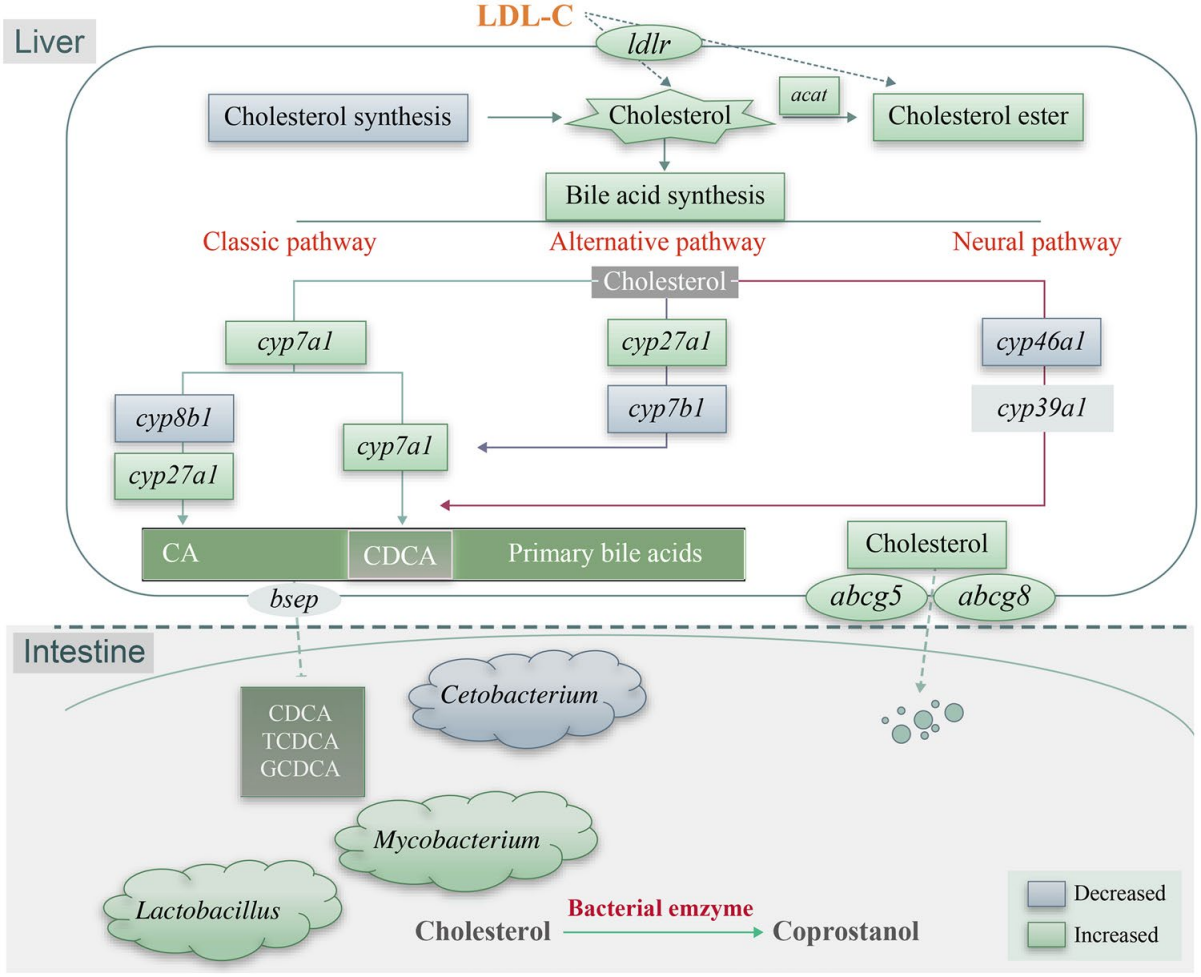
High cholesterol intake triggers protective adaptation via remodeling cholesterol turnover and gut microbiota composition. In response to high cholesterol, endogenous cholesterol synthesis pathway was inhibited, but bile acid synthesis and excretion (mainly by CDCA) and cholesterol excretion pathway were enhanced to reduce cholesterol accumulation in the liver. Additionally, the gut microbiota was reshaped to alleviate the high cholesterol/ bile acid stress in the high-cholesterol fish. LDL-C, low density lipoprotein cholesterol; *CA* cholic acid, *CDCA* chenodeoxycholic acid, *TCDCA* taurochenodeoxycholic acid, *GCDCA* glycochenodeoxycholic acid sodium salt, *acat* acetyl-CoA Acetyltransferase, *cyp7α1* cholesterol 7alpha-hydroxylase, *cyp27α1* cytochrome P450 family 27 subfamily A member 1, *cyp8b1* sterol 12a-hydroxylase, *cyp7b1* cytochrome P450 family 7 subfamily B member 1, *cyp46a1* cytochrome P450 family 46 subfamily A member 1, *abcg5/8* ATP-binding cassette, subfamily G, member 5/8, *bsep* bile salt export pump

Of note, animals lack the capability of catabolizing cholesterol, which can be used for the synthesis of bioactive substances, including bile acids (Goedeke and Fernández-Hernando 2012). Therefore, mammals mainly alleviate excess cholesterol accumulation in the body by triggering cholesterol conversion in the liver, a process which depends largely on the synthesis of bile acids (Russell 1992). The primary bile acids are synthesized from cholesterol exclusively in the liver mainly through two general pathways, i.e., the classic and alternative pathways (Bérard et al. 2004). The classic pathway produces approximately equal amounts of CA and CDCA (Bizuayehu et al. 2013). In the present study, the gallbladder was enlarged, and higher total bile acid content was found in the feces of fish fed with high-cholesterol diet. Interestingly, the CDCA synthesis pathway, but not CA synthesis was stimulated in the liver of fish fed with this diet. Accordingly, the high levels of CDCA, conjugated CDCA (TCDCA, GCDCA) and CDCA/CA ratio were observed in the intestine of fish fed with this high cholesterol diet. These results suggest that the classic bile acid synthesis pathway, especially CDCA synthesis, exerts an important role in response to high cholesterol intake in fish. Furthermore, given that the microbiome has an extensive metabolic repertoire and exerts roles in cholesterol and bile acid metabolism (Rowland et al. 2018), the changes in the intestinal microbiota were an adaptive response to high cholesterol intake. In the present study, we found that the abundance of *Lactobacillus* spp. and *Mycobacterium* spp., which are involved in cholesterol and/or bile acids catabolism (Gérard et al. 2007; Lye et al. 2010; Wang et al. 2012), were increased significantly in the intestine of fish fed with the high cholesterol diet. From data for Nile tilapia, it is considered that fish respond to high cholesterol intake by inhibiting endogenous cholesterol synthesis, enhancing cholesterol excretion as well as bile acid synthesis and excretion (mainly by CDCA), and enriching cholesterol breakdown-related bacteria in the intestine (Fig. 9). All these adaptive changes occurred to reduce excess cholesterol accumulation in tissues and alleviate the potential adverse effects caused by high cholesterol intake in fish.

### High cholesterol intake remodeled nutrient metabolism pattern to maintain energy production

Some studies have indicated that cholesterol has important functions in regulating energy metabolism in mammals (Chen et al. 2019). However, the effects of this regulation on nutrient metabolism patterns for lipid, carbohydrate and protein have not been well addressed for vertebrates. One of the obvious physiological phenotypes in the present study was the severe accumulation of lipid, especially cholesterol, accompanied by an increased number of lipid droplets in the liver of fish fed with the high cholesterol diet. The lipid droplet is the major site for cholesterol storage in many cells, and excess cholesterol is converted to the relatively inert storage form (cholesterol esters) (Farese and Walther 2009). Studies on mammals revealed that cholesterol accumulation led to the occurrence of autophagic flux (Li et al. 2020a). In fact, lipid droplet-associated autophagy, known as lipophagy, has been verified to be essential in lipid catabolism in mammals (Singh et al. 2009) and fish (Wang et al. 2018). In this study, fish reared on the high cholesterol diet increased protein expression of LC3-II, which is a marker of autophagy initiation. However, we found that, although the fish fed on high cholesterol diet contained many lipid droplet-enriched lysosomes in hepatocytes, the droplets were not broken down into smaller ones. Accordingly, the protein expression of Lal was reduced in fish fed with a high-cholesterol diet. These results indicated that the lipid droplet-degrading function of lysosomes may well be impaired in fish reared under cholesterol overload. Except for lipophagy, lipolysis and mitochondrial β-oxidation are the other two essential routes in lipid breakdown and cellular energy production (Han et al. 2021; Ning et al. 2016; Wang et al. 2019). However, the lipolysis-related key genes, *atgl* and *hsl,* were significantly downregulated. In addition, the mitochondrial [^14^C] palmitate β-oxidation efficiency, the protein expression of the key enzyme (Cpt1a) in the carnitine-dependent transport for mitochondrial β-oxidation and the related metabolites (including carnitine, acetylcarnitine, malonylcarnitine and β-hydroxybutyric acid) were all decreased in the fish fed with the high cholesterol diet. On the contrary, most genes related to lipogenesis, including *fasn*, *acly*, *accα*, *dgat* and *srepb1*, and the key regulatory factor m-Srebp1 protein were upregulated in fish fed with this diet. All these showed that lipid catabolism was systemically inhibited by high cholesterol intake. Overall, the results suggested that the fish fed with the high-cholesterol diet did not obtain enough energy sourced from lipid breakdown.

Lipid and carbohydrate metabolism are the main sources of mitochondrial oxidative phosphorylation for energy supply. Although fish are regarded as possessing a weak carbohydrate utilization capacity (Polakof et al. 2012), our previous studies indicated that glucose catabolism is stimulated when lipid catabolism is inhibited (Li et al. 2020b, 2020c). In addition, we found that glycolytic genes were upregulated, whereas gluconeogenic genes were downregulated in the liver of fish fed with the high-cholesterol diet in the present study. This might be associated with the cholesterol-stimulated Lxrα protein, which has been reported to participate in carbohydrate metabolism (Stulnig et al. 2002), by inducing metabolic switch towards aerobic glycolysis (Ikonomopoulou et al. 2021). However, our results indicated that the upregulation of glycolytic genes did not promote TCA cycle and oxidative phosphorylation in the liver of fish fed with the high-cholesterol diet. Furthermore, the [^14^C]-glucose tracking test confirmed the lower ^14^CO_2_ production from the fish in the high cholesterol group. This may well be connected to impaired mitochondrial function as the reduced mitochondria number and activated apoptosis in the liver. This is in accordance with some previous studies showing that cholesterol-induced cell apoptosis was mediated by a mitochondria-dependent pathway, which was connected to the loss of mitochondrial membrane potential and the release of cytochrome C (Green and Reed 1998; Leonarduzzi et al. 2007; Ly et al. 2003; Zhao et al. 2010). Additionally, high cholesterol and cholate diets induced hepatic insulin resistance in mice via inhibition of insulin receptor substrate-2 (IRS-2) expression (Matsuzawa et al. 2007). This is consistent with our findings that the fish fed with the high-cholesterol diet had weaker glucose clearance and lower hepatic glycogen content. This was associated with impaired insulin sensitivity and glycogen synthesis via inhibiting the activities of PI3k-AKT-GSK3*β* signaling pathway in the liver.

**Fig. 10:**
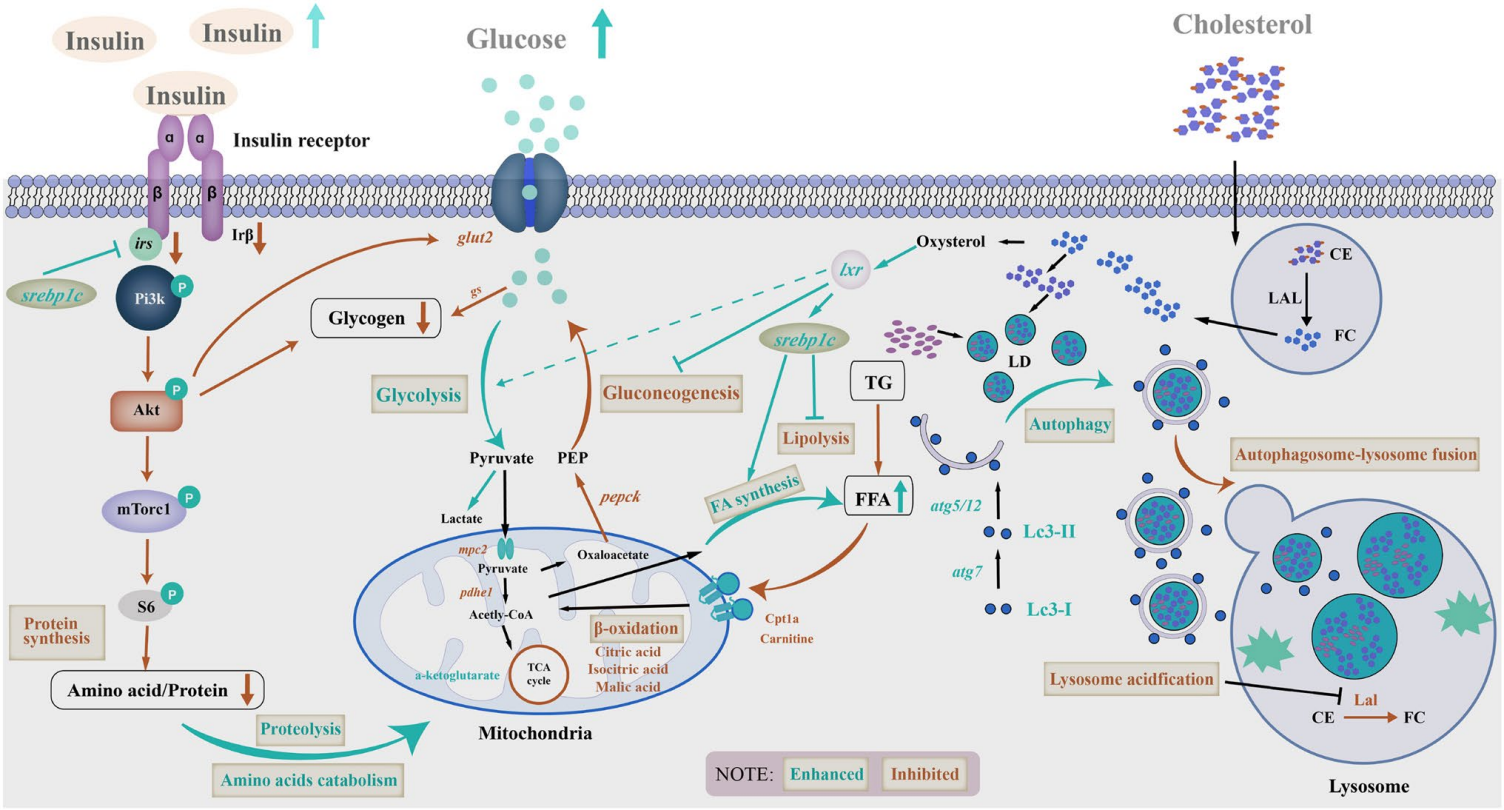
Hypothetical systemic metabolic responses to high cholesterol intake in the liver of Nile tilapia. In general, high cholesterol promotes lipid deposition by inhibiting triglyceride catabolism and fatty acid β-oxidation in the liver of fish. To maintain energy homeostasis, high cholesterol stimulated protein catabolism and oxidation for energy supply along with the inhibited insulin signaling pathway and glucose-derived oxidation phosphorylation. *TG* Triglyceride, *FFA* Free fatty acid, *FC* free cholesterol, *CE* cholesterol ester, *LD* lipid droplet, *glut2* glucose transporter protein 2, *irs* insulin receptor substrate, *gs* glycogen synthetase, *pepck* phosphoenolpyruvate carboxykinase, *pdhe1* pyruvate dehydrogenase E1 Subunit Alpha 1, *mpc2* mitochondrial pyruvate carrier 2, *atg5/7/12* autophagy-related genes 5/7/12, *Irβ* insulin receptor beta, *Pi3k* phosphoinositide 3-kinase, *Akt* a serine/threonine protein kinase, *mTorc1* target of rapamycin complex 1, *S6* ribosomal protein S6, *Srebp1c* sterol regulatory element binding protein 1c, *Lxr* liver x receptor, *Cpt1a* carnitine palmitoyl transferase 1a, *Lc3* microtubule-associated protein light chain 3, *Lal* lysosomal acid lipase

Given the fact that lipid and glucose catabolism were both inhibited, and there was no obvious growth retardation in the fish fed with the high-cholesterol diet, we hypothesized that protein catabolism should be stimulated for energy generation. Expectedly, although the dietary protein digestibility was significantly increased in the fish fed with the high cholesterol diet, the body protein content was decreased. Correspondingly, the amino acid contents were also decreased and the genes related to amino acids catabolism were upregulated in the fish. Recently, free cholesterol has been identified to serve as a nutrient to drive mTORC1 recruitment and activation through the conserved cholesterol-responsive motifs of SLC38A9 at the lysosomal surface (Castellano and Thelen 2017). In our study, the impaired lysosome function, especially the inhibited protein expression of Lal, which liberates free cholesterol from cholesterol ester, may well block the release of free cholesterol and then suppress the mTORC1 recruitment and activation. Evidently, the protein expressions of total mTorc1, p-mTorc1 and S6 were all largely reduced, and the [^14^C (U)]-AA catabolism was also significantly elevated in the fish fed with the high cholesterol diet. These results verified that the high cholesterol intake changed nutrient metabolism pattern to stimulate protein catabolism as the main energy source (Fig. 10).

## Conclusion

In summary, our data showed that dietary cholesterol supplementation at 0.8 to 3.2% caused body weight increase but excess cholesterol accumulation in tissues, especially in th liver accompanied by impaired liver function. The high cholesterol intake triggered protective adaptation via inhibiting endogenous cholesterol synthesis, promoting cholesterol esterification and efflux, CDCA synthesis and bile acids turnover and reshaping the gut microbiome to reduce cholesterol accumulation in fish. Moreover, the high cholesterol intake remodeled nutrient metabolism pattern by inhibiting lipid and glucose catabolism, but stimulating protein breakdown to maintain energy production. Therefore, although the high cholesterol intake promoted growth, it caused stress and metabolic disorders in fish. This is the first study illustrating the systemic metabolic responses of fish towards high cholesterol intake. The results obtained enlighten our understanding on the metabolic syndromes caused by high cholesterol intake or deposition in fish, and also help to optimize the usage of cholesterol in fish feed to promote the healthy growth of farmed fish.

## Materials and methods

### Experimental animals, diets and sample collection

Nile tilapia were obtained from Guangzhou Huihai Aquatic Technology Co., Ltd (Guangzhou, China). Before feeding trail, the fish were acclimated into an indoor-recirculating aquaculture system (200-L tanks) for two weeks. During the acclimatization period, the fish were fed with a commercial diet (Tongwei, Co. Ltd., Chengdu, China) containing 35% protein, 5% lipid and 0.05% cholesterol. For the experimental diets, casein and gelatin were supplemented as the protein sources; corn starch was used to provide the carbohydrate required whereas soybean oil served as the fat source. Five isonitrogenous and isoenergetic (approximately 39% protein, 32% carbohydrate and 6% fat) experimental diets were formulated with varying levels of cholesterol (0, 0.8, 1.6, 2.4 and 3.2%), in which the cholesterol-free diet was used in the control group (Supplementary Table S1). Cholesterol (> 99.0% purity, Glpbio, CA, USA) is not a kind of lipid ingredient for energy supply. The cholesterol contents of the experimental diets were determined using enzymatic assays (Allain et al. 1974). Briefly, the esterified cholesterol was hydrolyzed to free cholesterol by cholesterol esterase, and then the free cholesterol was oxidized to Δ4-cholesterone and H_2_O_2_ by cholesterol oxidase. Finally, the H_2_O_2_ was oxidized by peroxidase in the presence of 4-aminoantipyrine and phenol to form red quinones, which could be measured at 500 nm using a spectrophotometer. Yttrium oxide (99.99% purity; Shanghai Titan Scientific Co., Ltd., China) was added as an inert marker to the experimental diets for the measurement of protein digestibility. After the acclimation, 450 apparently healthy Nile tilapia, which were devoid of obvious signs of disease, with similar initial weights of 1.63 ± 0.03 g were randomly divided into five treatments (90 fish per treatment, 30 fish per tank, and three tanks per treatment). The experimental fish were randomly allocated to the dietary cholesterol levels and the control diet. The fish in each tank were fed twice daily at a feeding rate of 4% average body weight for eight weeks. Fish in each tank were bulk weighed each week to adjust the feeding amount. All the experimental diets were eaten quickly at each feeding time during the trial. During the experiment, water quality parameters were maintained at an optimum level for the survival and growth of Nile tilapia. Thus, the water temperature was 28.0 ± 1.0 °C, whereas dissolved oxygen, pH and total ammonia‑nitrogen ranged from 5.0 to 6.5 mg/L, 7.5 to 8.0 and below 0.2 mg/L, respectively.

At the end of the feeding trial, the fish in each tank were fasted overnight, euthanized (MS-222, Sigma, USA) and then individually weighed. Twelve fish from each treatment (four fish per tank) were individually weighed and sacrificed for sample collection. The liver and gallbladder of individual fish were weighed for th calculation of organ indices. In addition, serum, liver, gallbladder, intestine and feces samples were collected, immediately frozen into liquid nitrogen, and stored at − 80 °C until further analysis. Another six fish from each treatment (two fish per tank) were euthanized and killed for whole-body crude protein analysis.

### Proximate composition and nutrient digestibility measurement

The fish total protein in the whole body was analyzed using a semi-automatic Kjeldahl System (FOSS, Sweden). The total lipid in the liver was extracted using chloroform/methanol (2:1, v/v), and the lipid classes were separated from total lipids by thin-layer chromatography, as described previously (Lin et al. 2021). The concentrations of yttrium oxide in diets and feces were determined using atomic absorption spectrophotometry to calculate the protein apparent digestibility coefficient (ADC) (Reis et al. 2008) based on the yttrium oxide and protein contents in the diet and feces using the equation:

$$\mathrm{ADC}=100\mathrm{\%}\times \left[1-\left(\frac{{\mathrm{Y}}_{2}{\mathrm{O}}_{3}\,\mathrm{ in \,diet}}{{\mathrm{Y}}_{2}{\mathrm{O}}_{3}\,\mathrm{ in \,feces}} \times \frac{\mathrm{N\, in\, feces}}{\mathrm{N\, in\, diet}}\right)\right]$$

where ADC = apparent digestibility coefficient of protein; Y_2_O_3_ in diet = amount of yttrium oxide in the diet; Y_2_O_3_ in feces = amount of yttrium oxide in the feces; N in feces = protein content in the feces; and N in diet = protein content in the diet.

### Biochemical and histological analyses

The assay kits of total cholesterol (TC, Catalog: BC1985) and free cholesterol (FC, Catalog: BC1895) were purchased from Solarbio (Beijing, China). The assay kits of total bile acids (TBA, Catalog: E003-2-1), low-density lipoprotein cholesterol (LDL-C, Catalog: A113-1-1), free fatty acids (FFA, Catalog: A042-2-1), alanine aminotransferase (ALT, Catalog: C009-2-1), aspartate aminotransferase (AST, Catalog: C010-2-1), glucose (Catalog: F006-1-1) and glycogen (Catalog: A043-1-1) were obtained from Nanjing Jiancheng Bioengineering Institute (Nanjing, China). The enzyme-linked immunosorbent assay (ELISA) kit of insulin (Catalog: HB90131-QT) was acquired from Hengyuan Biotech Co., Ltd. (Shanghai, China). The TC, FC, TBA, LDL-C, FFA, ALT, AST, glucose and glycogen in different tissues were determined using specific commercial assay kits according to the manufacturer’s instructions. The insulin concentration in the serum was measured following the instructions.

The four liver tissues from each treatment were fixed in 4% paraformaldehyde solution and embedded in paraffin followed by neutral lipid staining, periodic acid–Schiff (PAS) staining and terminal deoxynucleotidyl transferase dUTP nick end label (TUNEL) staining according to standard procedures (Lillie and Fullmer 1976; Lu et al. 2019). Another four liver samples were immediately fixed using transmission electron microscopy (TEM) fixative (Catalog: G1102, Servicebio, Wuhan, China) overnight, and then processed as described previously (Lu et al. 2019). Ultra-thin sections were observed on a Hitachi HT7700 electron microscope (Hitachi High-Tech Corp., Tokyo, Japan).

### Glucose tolerance test

The glucose tolerance test was conducted using the method described previously (Liu et al. 2018). The 60 fish from the control and high cholesterol (1.6%) treatments have fasted overnight, and then intraperitoneally injected with D-glucose (500 mg/g fish body weight, 20% in saline solution). After injection, the blood was collected from the caudal vein of six fish from each treatment at every time point after 0, 0.5, 1, 3 and 6 h, and the serum glucose concentration was measured as described above.

### Quantitative real-time PCR and quantification of mitochondrial DNA

Total RNA from liver tissue was extracted using TRIzol reagent (Takara, Dalian, China) according to the manufacturer’s instructions. The quality and quantity of total RNA extracted were analyzed using a NanoDrop 2000 Spectrophotometer (Thermo Scientific, USA). cDNA synthesis was performed using the PrimeScript^™^ RT reagent kit following the manufacturer’s instructions. The quantitative real-time PCR (qPCR) was performed in a CFX96 Real-Time RCR detection system (Bio-Rad, USA) following ChamQ Universal SYBR qPCR Master Mix (Vazyme Biotech Co., Ltd., Nanjing, China) protocol. The qPCR reaction conditions were as follows: 95 °C for 2 min, followed by 40 cycles of 95 °C for 5 s and 60 °C for 15 s, and a final denaturation step from 60 to 95 °C for 30 s. Elongation factor 1 alpha (*EF1α*) and beta-actin (*β-actin*) were selected as the housekeeping genes. The housekeeping and target gene primers were listed in supplementary Table S2. A single qPCR product was evaluated using melting curve analysis at the end of each qPCR reaction. Primer amplification efficiency was between 90 and 105% where the correlation coefficient was more than 0.97 for each gene. The relative expression of target genes was measured using the 2^−∆∆Ct^ method.

Total genomic DNA was isolated from liver tissue using the Genomic DNA Extraction kit (TIANGEN) following the instructions. The NADH dehydrogenase 1 (*nd1*) and cytochrome b (*cytb*) genes located on mitochondrial DNA (mtDNA) were selected to measure the copy numbers of mtDNA relative to reference gene β-actin (Mt) using the qPCR method as described above. The specific primers for mtDNA are included in supplementary Table S2.

### Western blotting

Liver samples were collected from six fish from each treatment. Western blotting was conducted using RIPA buffer (NCM Biotech, China) according to the method described previously (Li et al. 2020b). The anti-CPT1a (1:800, 15184-1-AP, Proteintech), anti-LXRα/β (1:1000, sc-271064, Santa Cruz Biotechnology), anti-SREBP1c (1:1000, 66875-1-Ig, Proteintech), anti-LC3 (1:800, #4107, CST), anti-LAL (1:500, Custom Antibody Services from HuaAn Biotech, China), anti-IRβ (1:800, 20433-1-AP, Proteintech), anti-PI3K (1:800, CY6915, Abways), anti-p-PI3K (Tyr458/199) (1:800, #4228, CST), anti-AKT (1:800, #4091S, CST), anti-p-AKT (1:800, #4260S, CST), anti-mTORC1 (1:1000, #2972S, CST), anti-p-mTORC1 (1:1000, #2971S, CST), anti-S6 (1:1000, #2217S, CST), anti-p-S6 (1:800, AY0578, Abways), anti-GAPDH (1:3000, AB0036, Abways) and anti-α-Tubulin (1:1000, M1501-1, HuaAn Biotech) were used to measure the expressions of targeted proteins. The Western blotting images were obtained using an Odyssey CLx Imager (Licor, USA).

### Metabolic tracking test of ^14^C-labelled nutrients

After the eight-week trial, Nile tilapia from control and high cholesterol (1.6%) treatments were fasted overnight and then used for metabolic tracking of ^14^C-labelled nutrient. Six fish from each treatment were subjected to ^14^C-labelled nutrient tracking, including D-[1-^14^C]-glucose (Glu^∗^), [1-^14^C]-palmitic acid (PA^∗^) and L-[^14^C(U)]-amino acid mixture (AA^∗^) (PerkinElmer). The doses for intraperitoneal injection of ^14^C-labelled were based on our previous studies (Li et al. 2020c). To measure the ^14^CO_2_ released from the oxidation of different nutrients, the injected fish were immediately transferred to oxygen-saturated water in sealed bottles, which were connected to another glass bottle containing 1 mol/L potassium hydroxide (KOH) solution. The fish were digested with a strong lysate (HClO_4_ /30% H_2_O_2_, 2:1, v/v; 1:5, w/v) at 60 °C in a water bath for 6 h to ensure total retention of ^14^C in the whole body. The radioactivities of strong lysate and KOH solution (500 μl) were measured using the Tri-Carb 4910TR Liquid Scintillation Analyzer (PerkinElmer) with 2 ml of scintillation fluid (Ultima Gold XR; Packard, Conroe, TX, USA).

### Transcriptomic analysis

The nine liver samples were collected from each control and high cholesterol (1.6%) Nile tilapia treatments and prepared for transcriptomic analysis. A paired-end RNA-seq sequencing library was obtained using a Illumina HiSeq platform, and the clean reads were aligned to the Nile tilapia reference genome using TopHat2. The analysis of differentially expressed genes (DEGs) was performed based on a previous study (Lu et al. 2019).

### Metabolomics analysis

The twelve liver samples were collected from each control and high cholesterol (1.6%) Nile tilapia treatments for targeted metabolomics analysis on XploreMET platform (Metabo-Profile, Shanghai, China) according to our previous study (Li et al. 2022). The raw metabolomic data generated by UPLC-MS/MS were analyzed using iMAP (Metabo-Profile, Shanghai, China) platform for peak identification and quantification of each metabolite. The different biomarkers between the control and high cholesterol treatments were evaluated using orthogonal partial least squares discriminant analysis (OPLS-DA) by setting the thresholds of variable importance in projection (VIP) to > 1, fold change (FC) > 1 and *P* < 0.05.

### Intestinal total bacterial DNA extraction, illumina high-throughput sequencing of barcoded 16S rRNA genes

The intestinal contents of six fish from each treatment were collected. The total DNA of the bacterial community was extracted using DNA Extraction and Purification Kit (QIAGEN) according to the manufacturer’s instructions. The DNA quantity and quality were measured by a NanoDrop 2000 Spectrophotometer (Thermo, USA), and then used for the PCR amplification of the V4-V5 region of the bacterial 16S rRNA gene. The PCR reaction mixture was conducted following our previous study (Ma et al. 2018). PCR amplification was performed at conditions of 94 °C for 3 min, followed by 20 cycles at 95 °C for 1 min, 55 °C for 30 s, 72 °C for 30 s, and final elongation at 72 °C for 5 min using the forward and reverse primers 515F: 5′-GTGCCAGCMGCCGCGGTAA-3′ and 907R: 5′-CCGTCAATTCCTTTRAGTTT-3′, respectively. Unique barcodes were connected to the primer to distinguish PCR products. The purified PCR products were subjected to Illumina-based high-throughput sequencing (Majorbio Bio-Pharm Technology Co., Ltd., Shanghai, China).

The sequences were clustered as Operational Taxonomic Units (OTUs) at a 97% similarity level using Usearch (version 7.0), and chimeric sequences were identified and removed using UCHIME. Raw fast files were demultiplexed and quality-filtered with the criteria. Principal component analysis (PCA) was performed to distinguish different microbiome communities between the control and high cholesterol treatment. The bacterial taxonomic richness and diversity estimators were determined for each library in the Mothur website. The top 15 abundant bacteria species were compared using independent-samples *t* test (*P* ≤ 0.05 or *P* ≤ 0.01). The microbiome analyses were conducted on a bioinformation cloud computing platform (http://www.i-sanger.com).

### Statistical analyses

The data in Fig. 1A–I were analyzed by one-way analysis of variance (ANOVA) after the normality and homogeneity of variances by Levene’s test. Then, Tukey's multiple range test was used to determine significant differences (*P* < 0.05) among different high cholesterol groups. Other quantitative data were tested for normality using Shapiro–Wilk test, and homogeneity of variances was determined using Levene’s test. Then, an independent *t*-test was used to determine the significant differences (*P* < 0.05) on measured parameters between control and high cholesterol (1.6%) treatments using the SPSS Statistics 23.0 software (IBM, Michigan Avenue, USA). All results obtained are presented as mean values ± standard deviation (SD).

## Supplementary Information

Below is the link to the electronic supplementary material.

Supplementary file1 (DOCX 2547 KB)

## Data Availability

The datasets generated during and/or analysed during the current study are available from the corresponding author on reasonable request.

## References

[CR1] Allain CC, Poon LS, Chan CSG, Richmond W, Fu PC (1974). Enzymatic determination of total serum cholesterol. Clin Chem.

[CR2] Bérard AM, Dumon MF, Darmon M (2004). Dietary fish oil up-regulates cholesterol 7alpha-hydroxylase mRNA in mouse liver leading to an increase in bile acid and cholesterol excretion. FEBS Lett.

[CR3] Berger S, Raman G (2015). Dietary cholesterol and cardiovascular disease: a systematic review and meta-analysis. Am J Clin Nutr.

[CR4] Bizuayehu TT, Fernandes JM, Johansen SD, Babiak I (2013). Characterization of novel precursor miRNAs using next generation sequencing and prediction of miRNA targets in Atlantic halibut. PLoS ONE.

[CR5] Castellano BM, Thelen AM (2017). Lysosomal cholesterol activates mTORC1 via an SLC38A9-Niemann-Pick C1 signaling complex. Science.

[CR6] Chen L, Chen XW, Huang X, Song BL, Wang Y, Wang Y (2019). Regulation of glucose and lipid metabolism in health and disease. Sci China Life Sci.

[CR7] Chen P, Zhu YP, Wu XF, Gu X, Xue M, Liang XF (2022). Metabolic adaptation to high starch diet in largemouth bass (*Micropterus salmoides*) was associated with the restoration of metabolic functions via inflammation, bile acid synthesis and energy metabolism. Br J Nutr.

[CR8] Deng J, Mai K, Ai Q, Zhang W, Wang X, Tan B, Xu W, Liufu Z, Ma H (2010). Interactive effects of dietary cholesterol and protein sources on growth performance and cholesterol metabolism of Japanese flounder (*Paralichthys olivaceus*). Aquacult Nutr.

[CR9] Dietschy JM (1984). Regulation of cholesterol metabolism in man and in other species. Klin Wochenschr.

[CR10] Farese RV, Walther TC (2009). Lipid droplets finally get a little R-E-S-P-E-C-T. Cell.

[CR11] Gérard P, Lepercq P, Leclerc M, Gavini F, Raibaud P, Juste C (2007). Bacteroides sp. strain D8, the first cholesterol-reducing bacterium isolated from human feces. Appl Environ Microbiol.

[CR12] Goedeke L, Fernández-Hernando C (2012). Regulation of cholesterol homeostasis. Cell Mol Life Sci.

[CR13] Green DR, Reed JC (1998). Mitochondria and apoptosis. Science.

[CR14] Guyon R, Rakotomanga M, Azzouzi N, Coutanceau JP, Bonillo C, D'Cotta H, Pepey E, Soler L, Rodier-Goud M, D'Hont A, Conte MA, van Bers NE, Penman DJ, Hitte C, Crooijmans RP, Kocher TD, Ozouf-Costaz C, Baroiller JF, Galibert F (2012). A high-resolution map of the Nile tilapia genome: a resource for studying cichlids and other percomorphs. BMC Genom.

[CR15] Han C, Wang J, Li L, Wang L, Zhang Z (2009). The role of LXR alpha in goose primary hepatocyte lipogenesis. Mol Cell Biochem.

[CR16] Han CC, Wang JW, Pan ZX, Tang H, Xiang SX, Wang J, Li L, Xu F, Wei SH (2013). Effect of cholesterol on lipogenesis and VLDL-TG assembly and secretion in goose primary hepatocytes. Mol Cell Biochem.

[CR17] Han SL, Qian YC, Limbu SM, Wang J, Chen LQ, Zhang ML, Du ZY (2021). Lipolysis and lipophagy play individual and interactive roles in regulating triacylglycerol and cholesterol homeostasis and mitochondrial form in zebrafish. Biochim Biophys Acta Mol Cell Biol Lipids.

[CR18] Hao M, Head WS, Gunawardana SC, Hasty AH, Piston DW (2007). Direct effect of cholesterol on insulin secretion: a novel mechanism for pancreatic beta-cell dysfunction. Diabetes.

[CR19] He C, Jia X, Zhang L, Gao F, Jiang W, Wen C, Chi C, Li X, Jiang G, Mi H, Liu W, Zhang D (2021). Dietary berberine can ameliorate glucose metabolism disorder of *Megalobrama amblycephala* exposed to a high-carbohydrate diet. Fish Physiol Biochem.

[CR20] Ikonomopoulou MP, Lopez-Mancheño Y, Novelle MG, Martinez-Uña M, Gangoda L, Pal M, Costa-Machado LF, Fernandez-Marcos PJ, Ramm GA, Fernandez-Rojo MA (2021). LXR stimulates a metabolic switch and reveals cholesterol homeostasis as a statin target in Tasmanian devil facial tumor disease. Cell Rep.

[CR21] Jobling M (2012). National Research Council (NRC): nutrient requirements of fish and shrimp. Aquacult Int.

[CR22] Kortner TM, Gu J, Krogdahl Å, Bakke AM (2013). Transcriptional regulation of cholesterol and bile acid metabolism after dietary soyabean meal treatment in Atlantic salmon (*Salmo salar* L.). Br J Nutr.

[CR23] Leaver MJ, Villeneuve LA, Obach A, Jensen L, Bron JE, Tocher DR, Taggart JB (2008). Functional genomics reveals increases in cholesterol biosynthetic genes and highly unsaturated fatty acid biosynthesis after dietary substitution of fish oil with vegetable oils in Atlantic salmon (*Salmo salar*). BMC Genom.

[CR24] Leonarduzzi G, Poli G, Sottero B, Biasi F (2007). Activation of the mitochondrial pathway of apoptosis by oxysterols. Front Biosci.

[CR25] Li K, Deng Y, Deng G, Chen P, Wang Y, Wu H, Ji Z, Yao Z, Zhang X, Yu B, Zhang K (2020). High cholesterol induces apoptosis and autophagy through the ROS-activated AKT/FOXO1 pathway in tendon-derived stem cells. Stem Cell Res Ther.

[CR26] Li LY, Li JM, Ning LJ, Lu DL, Luo Y, Ma Q, Limbu SM, Li DL, Chen LQ, Lodhi IJ, Degrace P, Zhang ML, Du ZY (2020). Mitochondrial fatty acid β-oxidation inhibition promotes glucose utilization and protein deposition through energy homeostasis remodeling in fish. J Nutr.

[CR27] Li LY, Lv HB, Jiang ZY, Qiao F, Chen LQ, Zhang ML, Du ZY (2020). Peroxisomal proliferator-activated receptor α-b deficiency induces the reprogramming of nutrient metabolism in zebrafish. J Physiol.

[CR28] Li RX, Zhou WH, Ren J, Wang J, Qiao F, Zhang ML, Du ZY (2022). Dietary sodium lactate promotes protein and lipid deposition through increasing energy supply from glycolysis in Nile tilapia (*Oreochromis niloticus*). Aquaculture.

[CR29] Lillie RD, Fullmer H (1976). Histopathological technique and practical histochemistry.

[CR30] Lin Z, Wang X, Bu X, Jia Y, Shi Q, Du Z, Qin J, Chen L (2021). Dietary phosphatidylcholine affects growth performance, antioxidant capacity and lipid metabolism of Chinese mitten crab (*Eriocheir sinensis*). Aquaculture.

[CR31] Liu CZ, He AY, Ning LJ, Luo Y, Li DL, Zhang ML, Chen LQ, Du ZY (2018). Leptin selectively regulates nutrients metabolism in Nile tilapia fed on high carbohydrate or high fat diet. Front Endocrinol (lausanne).

[CR32] Lu DL, Ma Q, Wang J, Li LY, Han SL, Limbu SM, Li DL, Chen LQ, Zhang ML, Du ZY (2019). Fasting enhances cold resistance in fish through stimulating lipid catabolism and autophagy. J Physiol.

[CR33] Luo J, Yang H, Song B-L (2020). Mechanisms and regulation of cholesterol homeostasis. Nat Rev Mol Cell Biol.

[CR34] Ly JD, Grubb DR, Lawen A (2003). The mitochondrial membrane potential (deltapsi(m)) in apoptosis; an update. Apoptosis.

[CR35] Lye HS, Rusul G, Liong MT (2010). Removal of cholesterol by lactobacilli via incorporation and conversion to coprostanol. J Dairy Sci.

[CR36] Ma Q, Li L-Y, Le J-Y, Lu D-L, Qiao F, Zhang M-L, Du Z-Y, Li D-L (2018). Dietary microencapsulated oil improves immune function and intestinal health in Nile tilapia fed with high-fat diet. Aquaculture.

[CR37] Matsuzawa N, Takamura T, Kurita S, Misu H, Ota T, Ando H, Yokoyama M, Honda M, Zen Y, Nakanuma Y, Miyamoto K, Kaneko S (2007). Lipid-induced oxidative stress causes steatohepatitis in mice fed an atherogenic diet. Hepatology.

[CR38] Min HK, Kapoor A, Fuchs M, Mirshahi F, Zhou H, Maher J, Kellum J, Warnick R, Contos MJ, Sanyal AJ (2012). Increased hepatic synthesis and dysregulation of cholesterol metabolism is associated with the severity of nonalcoholic fatty liver disease. Cell Metab.

[CR39] Ning LJ, He AY, Li JM, Lu DL, Jiao JG, Li LY, Li DL, Zhang ML, Chen LQ, Du ZY (2016). Mechanisms and metabolic regulation of PPARα activation in Nile tilapia (*Oreochromis niloticus*). Biochim Biophys Acta.

[CR40] Polakof S, Panserat S, Soengas JL, Moon TW (2012). Glucose metabolism in fish: a review. J Comp Physiol B.

[CR41] Reis PA, Valente LMP, Almeida CMR (2008). A fast and simple methodology for determination of yttrium as an inert marker in digestibility studies. Food Chem.

[CR42] Rowland I, Gibson G, Heinken A, Scott K, Swann J, Thiele I, Tuohy K (2018). Gut microbiota functions: metabolism of nutrients and other food components. Eur J Nutr.

[CR43] Russell DW (1992). Cholesterol biosynthesis and metabolism. Cardiovasc Drugs Ther.

[CR44] Sallam T, Jones MC, Gilliland T, Zhang L, Wu X, Eskin A, Sandhu J, Casero D, Vallim TQ, Hong C, Katz M, Lee R, Whitelegge J, Tontonoz P (2016). Feedback modulation of cholesterol metabolism by the lipid-responsive non-coding RNA LeXis. Nature.

[CR45] Sealey WM, Craig SR, Gatlin DM (2001). Dietary cholesterol and lecithin have limited effects on growth and body composition of hybrid striped bass (*Morone chrysops* × *M. saxatilis*). Aquacult Nutr.

[CR46] Sheridan MA (1988). Lipid dynamics in fish: aspects of absorption, transportation, deposition and mobilization. Comp Biochem Physiol B.

[CR47] Singh R, Kaushik S, Wang Y, Xiang Y, Novak I, Komatsu M, Tanaka K, Cuervo AM, Czaja MJ (2009). Autophagy regulates lipid metabolism. Nature.

[CR48] Stranberg TE, Salomaa V, Vanhanen H, Miettinen TA (1996). Associations of fasting blood glucose with cholesterol absorption and synthesis in nondiabetic middle-aged men. Diabetes.

[CR49] Stulnig TM, Steffensen KR, Gao H, Reimers M, Dahlman-Wright K, Schuster GU, Gustafsson JA (2002). Novel roles of liver X receptors exposed by gene expression profiling in liver and adipose tissue. Mol Pharmacol.

[CR50] Tang T, Hu Y, Peng M, Chu W, Hu Y, Zhong L (2019). Effects of high-fat diet on growth performance, lipid accumulation and lipid metabolism-related MicroRNA/gene expression in the liver of grass carp (*Ctenopharyngodon idella*). Comp Biochem Physiol B Biochem Mol Biol.

[CR51] Tao YF, Qiang J, Bao JW, Chen DJ, Yin GJ, Xu P, Zhu HJ (2018). Changes in physiological parameters, lipid metabolism, and expression of microRNAs in genetically improved farmed tilapia (*Oreochromis niloticus*) with fatty liver induced by a high-fat diet. Front Physiol.

[CR52] Twibell RG, Wilson RP (2004). Preliminary evidence that cholesterol improves growth and feed intake of soybean meal-based diets in aquaria studies with juvenile channel catfish, *Ictalurus punctatus*. Aquaculture.

[CR53] Wang Z, Zeng X, Mo Y, Smith K, Guo Y, Lin J (2012). Identification and characterization of a bile salt hydrolase from *Lactobacillus salivarius* for development of novel alternatives to antibiotic growth promoters. Appl Environ Microbiol.

[CR54] Wang J, Han SL, Li LY, Lu DL, Limbu SM, Li DL, Zhang ML, Du ZY (2018). Lipophagy is essential for lipid metabolism in fish. Sci Bull.

[CR55] Wang J, Han SL, Lu DL, Li LY, Limbu SM, Li DL, Zhang ML, Du ZY (2019). Inhibited lipophagy suppresses lipid metabolism in zebrafish liver cells. Front Physiol.

[CR56] Xu R, Li M, Wang T, Zhao YW, Shan CJ, Qiao F, Chen LQ, Zhang WB, Du ZY, Zhang ML (2022). Bacillus amyloliquefaciens ameliorates high-carbohydrate diet-induced metabolic phenotypes by restoration of intestinal acetate-producing bacteria in Nile Tilapia. Br J Nutr.

[CR57] Yue S, Li J, Lee SY, Lee HJ, Shao T, Song B, Cheng L, Masterson TA, Liu X, Ratliff TL, Cheng JX (2014). Cholesteryl ester accumulation induced by PTEN loss and PI3K/AKT activation underlies human prostate cancer aggressiveness. Cell Metab.

[CR58] Yun B, Mai K, Zhang W, Xu W (2011). Effects of dietary cholesterol on growth performance, feed intake and cholesterol metabolism in juvenile turbot (*Scophthalmus maximus* L.) fed high plant protein diets. Aquaculture.

[CR59] Zhao YF, Wang L, Lee S, Sun Q, Tuo Y, Wang Y, Pei J, Chen C (2010). Cholesterol induces mitochondrial dysfunction and apoptosis in mouse pancreatic beta-cell line MIN6 cells. Endocrine.

[CR60] Zhu T, Corraze G, Plagnes-Juan E, Quillet E, Dupont-Nivet M, Skiba-Cassy S (2018). Regulation of genes related to cholesterol metabolism in rainbow trout (*Oncorhynchus mykiss*) fed a plant-based diet. Am J Physiol Regul Integr Comp Physiol.

[CR61] Zhu T, Mai K, Xu W, Ai Q (2018). Effect of dietary cholesterol and phospholipids on feed intake, growth performance and cholesterol metabolism in juvenile turbot (*Scophthalmus maximus* L.). Aquaculture.

