# Supplementary material for: High cholesterol intake remodels cholesterol turnover and energy homeostasis in Nile tilapia (Oreochromis niloticus)
Source: Mar Life Sci Technol. 2023 Feb 16;5(1):56–74. doi: 10.1007/s42995-022-00158-7 (PMC10077235; doi:10.1007/s42995-022-00158-7)
Supplement: Supplementary file 1 — Supplementary file1 (DOCX 2547 KB) [file 42995_2022_158_MOESM1_ESM.docx]

**Supplementary Information**

**High cholesterol intake remodels cholesterol turnover and energy homeostasis in Nile tilapia (*Oreochromis niloticus*)**

**Rui-Xin Li^1^, Ling-Yun Chen^1^, Samwel M. Limbu^2^, Yu-Cheng Qian^1^, Wen-Hao Zhou^1^, Li-Qiao Chen^1^, Yuan Luo^1^, Fang Qiao^1^, Mei-Ling Zhang^1^, Zhen-Yu Du^1^***

*^1^ LANEH, School of Life Sciences,* *East China Normal University, 200241 Shanghai, China*

*^2^ Department of Aquaculture Technology, School of Aquatic Sciences and Fisheries Technology, University of Dar es Salaam, P. O. Box 60091, Dar es Salaam, Tanzania*

**Corresponding author：**

Prof. Zhen-Yu Du, School of Life Sciences, East China Normal University, 500 Dong Chuan Road, 200241 Shanghai, China.

E-mail address: zydu@bio.ecnu.edu.cn (Z.-Y. Du).

**List of content:**

**Supplementary Table S1：**Formulation and proximate analysis of the experimental diets.

**Supplementary Table S2：**The primer sequences applied for quantitative real-time PCR analysis in Nile tilapia.

**Supplementary Figure S1:** TC and FC content in the adipose, muscle and intestine tissues of Nile tilapia fed C and HC diets for eight weeks.

**Supplementary Figure S2:** Visceral mass and liver appearance, protein expressions of Cpt1a, Lxr and Srebp1c, Lc3 I/II, Beclin and Lal of Nile tilapia fed C and HC diets for eight weeks.

**Supplementary Figure S3:** Insulin signaling (A), glucose metabolism (B) and mTORC1 signaling pathway (C) of Nile tilapia fed C and HC diets for eight weeks.

**Table S1** Formulation and proximate analysis of the experimental diets

| Ingredients (g/kg) | Control | 0.8% CHOL | 1.6% CHOL | 2.4% CHOL | 3.2% CHOL |
| --- | --- | --- | --- | --- | --- |
| Casein | 320 | 320 | 320 | 320 | 320 |
| Gelatin | 90 | 90 | 90 | 90 | 90 |
| Corn starch | 320 | 320 | 320 | 320 | 320 |
| Soybean oil | 60 | 60 | 60 | 60 | 60 |
| Vitamin^1^ | 15 | 15 | 15 | 15 | 15 |
| Minerals^2^ | 25 | 25 | 25 | 25 | 25 |
| CMC | 24.75 | 24.75 | 24.75 | 24.75 | 24.75 |
| Cellulose | 125 | 117 | 109 | 101 | 93 |
| Choline chloride | 5 | 5 | 5 | 5 | 5 |
| Ca(H_2_PO_4_)_2_ | 10 | 10 | 10 | 10 | 10 |
| Dimethly-β-propiothetin | 0.25 | 0.25 | 0.25 | 0.25 | 0.25 |
| Yttrium oxide | 5 | 5 | 5 | 5 | 5 |
| Cholesterol | 0 | 8 | 16 | 24 | 32 |
| Total | 1000 | 1000 | 1000 | 1000 | 1000 |
| Proximate Composition (%, dry matter) | | | |  |  |
| Dry matter | 90.2 | 90.3 | 91.2 | 90.8 | 91.5 |
| Crude protein | 38.5 | 39.1 | 38.1 | 37.9 | 38.3 |
| Crude lipid | 6.1 | 6.7 | 7.5 | 8.2 | 9.1 |
| Cholesterol | 0.01 | 0.7 | 1.5 | 2.4 | 3.3 |
| Ash | 5.8 | 6.3 | 6.2 | 5.7 | 6.4 |

^1^ Vitamin premix, (mg or IU/kg): 500,000 I.U. (international units) Vitamin A, 50,000 I.U. Vitamin D3, 2500 mg Vitamin E, 1000 mg Vitamin K3, 5000 mg Vitamin B1, 5000 mg Vitamin B2, 5000 mg Vitamin B6, 5000μg Vitamin B12, 25,000 mg Inositol, 10,000 mg Pantothenic acid, 100,000 mg Cholin, 25,000 mg Niacin, 1000 mg Folic acid, 250 mg Biotin, 10,000 mg Vitamin C.

^2^ Mineral premix, (g/kg): 314.0 g CaCO_3_; 469.3 KH_2_PO_4_; 147.4 g MgSO_4_·7H_2_O; 49.8 g NaCl; 10.9 g Fe(II) gluconate; 3.12 g MnSO_4_·H_2_O; 4.67 g ZnSO_4_·7H_2_O; 0.62 g CuSO_4_·5H_2_O; 0.16 g; 0.08 g CoCl_2_·6H_2_O; 0.06 g NH_4_ molybdate; 0.02 g NaSeO_3_

**Table S2** The primer sequences applied for quantitative real-time PCR analysis in Nile tilapia

| Gene | Forward primer (5’-to 3’-) | Reverse primer (5’-to 3’-) | Product length (bp) | Amplification efficiency (%) | Accession No. |
| --- | --- | --- | --- | --- | --- |
| *bcl2* | ATCGCAGACTGGATGACGGAGTAT | TCTGTCTGTCGTACAGCTCCACAA | 100 | 97 | XM_003437902.4 |
| *caspase 3* | GGAGTGGACGATACAGACGCAAA | TGAAGCTGTGTGACTGGGGCTT | 109 | 93 | NM_001282894.1 |
| *caspase 9* | ATACTTGAGGAAAACGCTGCCACT | GAACCAGGCATTTGTTTGTAGAGC | 110 | 96 | XM_003455320.4 |
| *p53* | CGCAGACCTATCCTCACCAT | AGGAGGTGGAGCACTCTTTC | 183 | 101 | XM_025905404.1 |
| *atgl* | GACACATGCTGCAAAGCACT | ACCAGGACGTTTTCTCCGTC | 103 | 95 | XM_003440346.5 |
| *hsl* | AGTTCACTCCAGCCATTCGG | TGGCTGCTACCCCTATTCCT | 105 | 97 | XM_005463937.4 |
| *mgl* | GGGCTCCATCGAGTCCAAAT | AATGATACTCGCATCCCGCC | 99 | 91 | XM_005478351.4 |
| *fasn* | TCATCCAGCAGTTCACTGGCATT | TGATTAGGTCCACGGCCACA | 102 | 99 | GU433188 |
| *accɑ* | TAGCTGAAGAGGAGGGTGCAAGA | AACCTCTGGATTGGCTTGAACA | 110 | 98 | XM_005471970 |
| *acly* | AAAAGCTTTGATGAGCTTGGGG | TACAGTGGGAGGAGGCAACTCTT | 102 | 98 | XM_003442027 |
| *dgat* | GCTTGAATTCTGTCACCCTGAAGA | ACCTGCTTGTAGGCGTCGTTCT | 115 | 101 | XM_003458972 |
| *srebp1* | TGCAGCAGAGAGACTGTATCCGA | ACTGCCCTGAATGTGTTCAGACA | 102 | 98 | XM_005471970 |
| *lxrɑ* | GTAAGGTGTTTGATGGGGC | ATTATGAGGGGGGACGG | 143 | 91 | XM_005455718.4 |
| *atg5* | ACAGCGTCTTACCCTGGAGCA | TCACAGAGCTGGATGGGCAGT | 84 | 95 | XM_003450274.5 |
| *atg7* | TCTCTCAGACCACTCTGTCCC | AGCAGCATTCACCACTAGCTT | 161 | 103 | XM_003454570.5 |
| *atg12* | TCATATCTCGCTTCCTCAAGC | CCCTACTTCTTGATCCGGTGA | 92 | 98 | XM_025911479.1 |
| *p62* | CCCTTCTAAACCTGCTGCTGA | TCACCTTGGTCCGTTGGC | 158 | 99 | XM_005463795.4 |
| *lanmp1* | CTGGTTTTGACACAGACGCAA | ATGTAGGAGTAGCCCAGCGT | 179 | 102 | XM_003445782.5 |
| *insr* | TTCAGCTGCCACCACGT | TCATCAGCTCCATCACCACCA | 79 | 93 | KC517071.1 |
| *ins-2* | ATGGCCAAGGGAGAGAGGAT | ATCAACTCTCAAGCAGCCCC | 287 | 96 | XM_005449960.4 |
| *glut2* | CATTGGCATTCTAATCAGCCAGGT | TTGTAATATTGCTGGCGCTCCA | 106 | 96 | XM_003442884.5 |
| *gs* | CCTCACTCTGCGCTGTTATTC | CAGCGGCATGCCTTCAGTTT | 100 | 94 | XM_013276796.3 |
| *gsk3β* | GACGAGAACTGCCGATGCTCTT | CGTGAGGAGGAATGAGGATTGAGT | 87 | 102 | XM_003454610.5 |
| *pk* | CAGCATAATCTGCACCATCGGT | ATGAGAGAAGTTAAGACGGGCGA | 100 | 98 | XM_005472621.3 |
| *hk* | CACTGAGCTCAAGGATGACCA | CTCGGGCGTGTCGTAGATTT | 316 | 110 | XM_003454508 |
| *g6pase* | AGACCTTATTGGTGGGTTCACGA | CTGAAGGACTTCCTGGTCCAGTTT | 106 | 99 | XM_003448671.4 |
| *pepck* | TGGAAGAACAAACCTTGGCG | TGGGTCAATAATGGGACACTGTCT | 99 | 100 | XM_003448375 |
| *pdhea-ɑ* | ATGAGCGATCCTGGCGTTAG | AAGGGGTTGGGTGGGTTATG | 322 | 99 | XM_003446838.5 |
| *cs* | AGCACCACAGTTTACCAG | AGTGTTGACAAACCCAGA | 218 | 91 | XM_003438897 |
| *idh* | ACGCATCGCTGAGTACGCCTT | AGACCGTCTGACATCCGCATGA | 99 | 106 | XM_003437590.5 |
| *β-actin* | AGCCTTCCTTCCTTGGTATGGAAT | TGTTGGCGTACAGGTCCTTACG | 102 | 93 | KJ126772.1 |
| *ef1a* | ATCAAGAAGATCGGCTACAACCCT | ATCCCTTGAACCAGCTCATCTTGT | 109 | 103 | KJ123689.1 |
| *nd1* | CCCAAACATCGTAGGCCCTT | GGGTCGAACGGGCTCTTTTA | 82 | 94 | 8677324 |
| *cytb* | GCCTAACCTTCCGCCCTATC | TTGTTTTCTAGCCAGCCGGT | 180 | 98 | 8677323 |
| *β-actin (Mt)* | TGCCTTACCTGCACTTGT | GCATTAGAGTGAGGCTGATTGA | 159 | 95 | KJ126772.1 |

**Fig. S1**


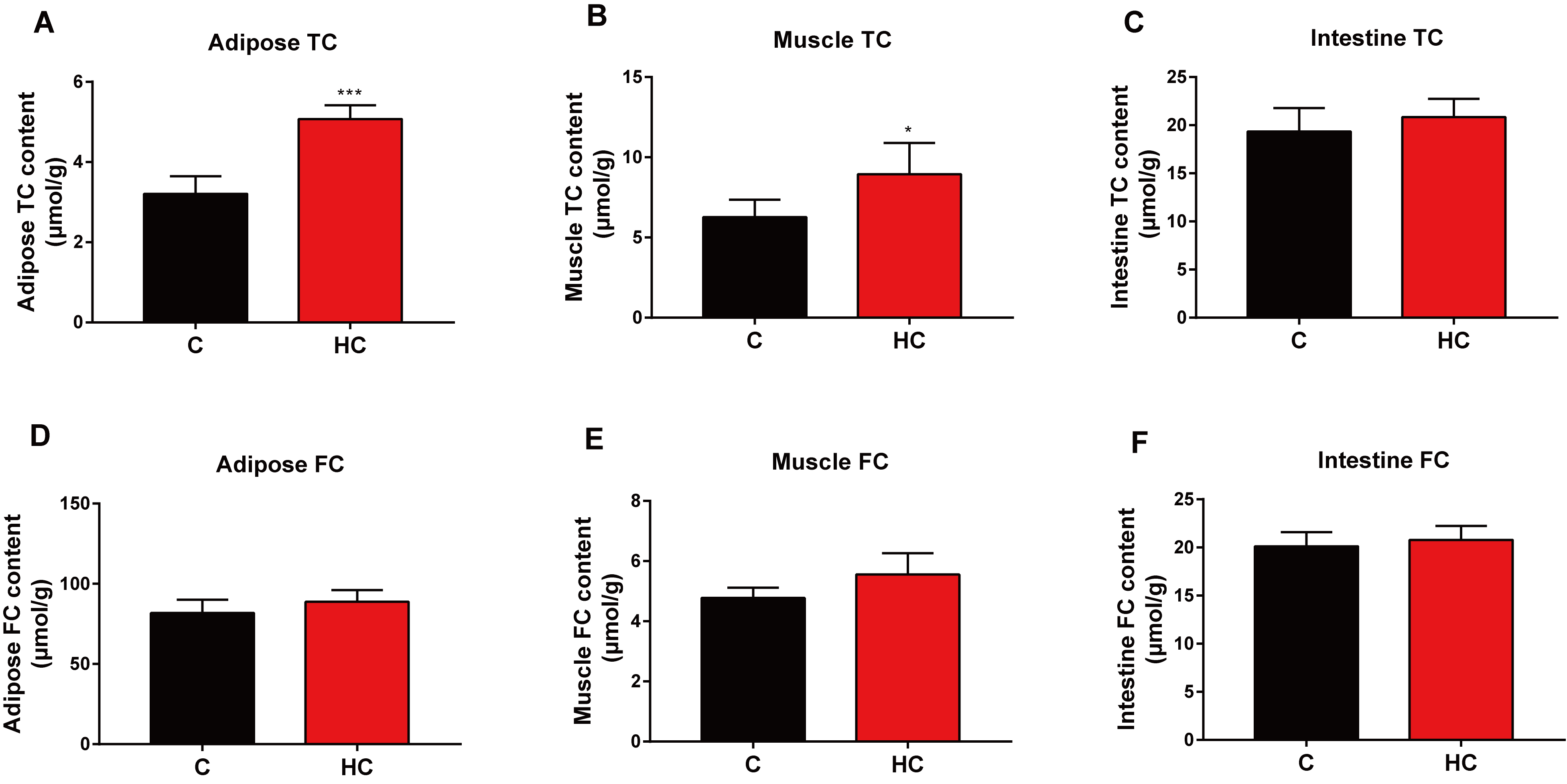


**Fig. S1** TC and FC content in adipose (A & D), muscle (B & E) and intestine (C & F) tissues of Nile tilapia fed C and HC diets for eight weeks. Data are represented as mean ± SD (TC and FC content in adipose, muscle and intestine: n = 6). **P* < 0.05, ***P* < 0.01 and ****P* < 0.001 significance (independent *t-*test) between HC and C groups. C, control diet; HC, high cholesterol diet.

**Fig. S2**


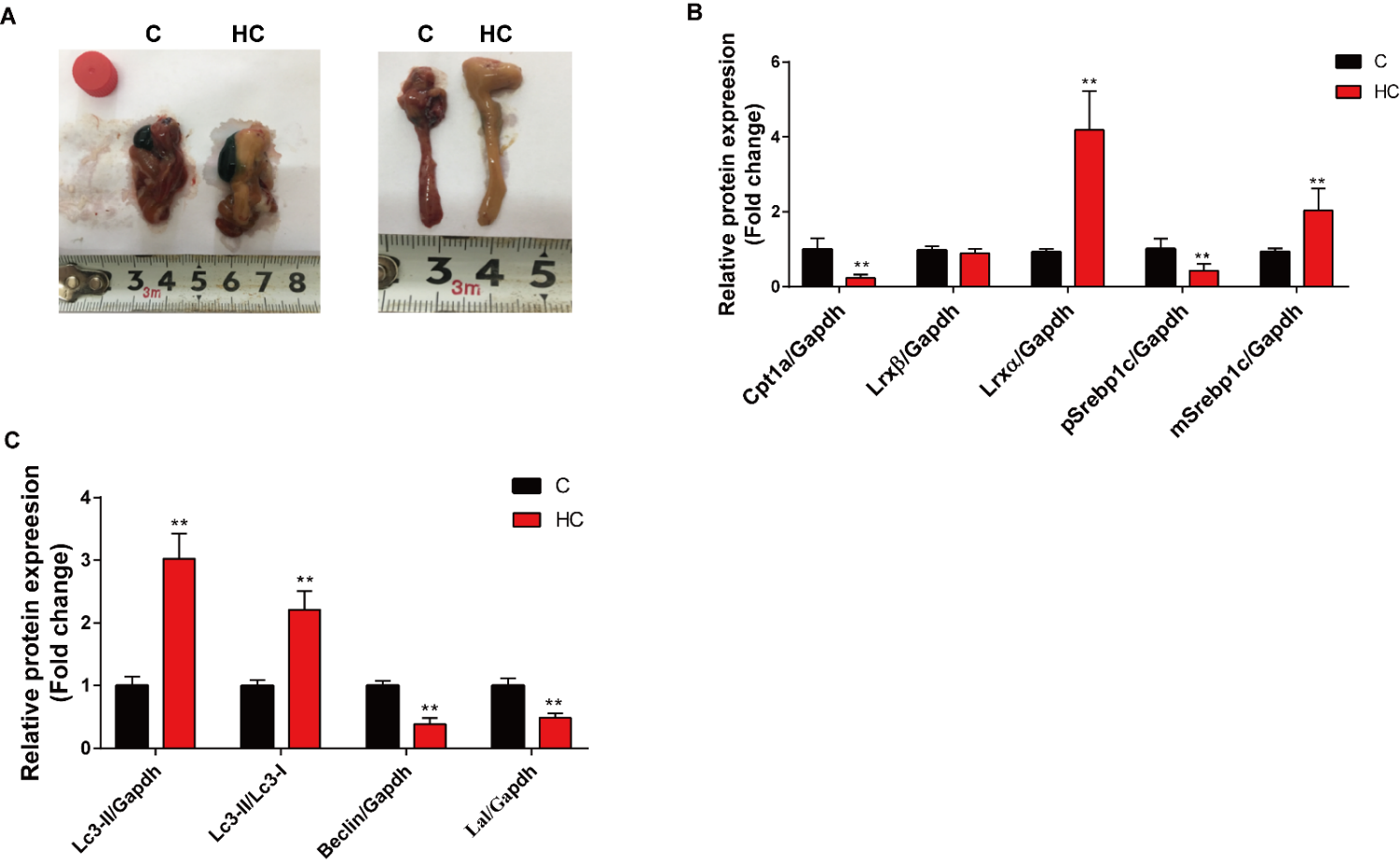


**Fig. S2** Visceral mass and liver appearance (A), protein expression of Cpt1a, Lxr and Srebp1c (B), Lc3 I/II, Beclin and Lal (C) of Nile tilapia fed C and HC diets for eight weeks. Data are represented as mean ± SD (Protein expression: n = 6). **P* < 0.05 and ***P* < 0.01 indicate significant difference (2-tailed independent *t* test) between HC and C groups. C, control diet; HC, high cholesterol diet; Cpt1a, carnitine palmitoyl transferase 1a; Lxrα/β, liver x receptorα/β; p/m-Srebp1c, precursor/mature form of sterol regulatory element binding protein 1c; Gapdh, glyceraldehyde-3-phosphate dehydrogenase; Lc3, microtubule-associated protein 1A/1B-light chain 3; Lal, lysosomal acid lipase.

**Fig. S3**





**Fig. S3** Insulin signaling (A), glucose metabolism (B) and mTORC1 signaling pathway (C) of Nile tilapia fed C and HC diets for eight weeks. Data are represented as mean ± SD (protein expression: n = 6; transcriptome heatmap: n = 9). **P* < 0.05 and ***P* < 0.01 indicate significant difference (independent *t*-test) between HC and C groups. C, control diet; HC, high cholesterol diet; *Irβ*, insulin receptor beta; Pi3k, phosphoinositide 3-kinase; Akt, serine/threonine kinase; mTorc1, target of rapamycin complex 1; S6, ribosomal protein S6; Gapdh, glyceraldehyde-3-phosphate dehydrogenase; *pk*, pyruvate kinase; *pfk*, phosphofructokinase; *idha*, lactate dehydrogenase-A; *adh*, alcohol dehydrogenase; *mpc2*, mitochondrial pyruvate carrier 2; *pdhe1*, pyruvate dehydrogenase E1 subunit; *g6pase*, glucose-6-phosphatase; *fbp*, fructose-bisphosphatase; *pepck*, phosphoenolpyruvate carboxykinase; *mdh*, malate dehydrogenase; *sdh*, succinate dehydrogenase; *idh*, isocitrate dehydrogenase.
